# Biologically active constituents of the secretome of human W8B2^+^ cardiac stem cells

**DOI:** 10.1038/s41598-018-19855-4

**Published:** 2018-01-25

**Authors:** Shuai Nie, Xin Wang, Priyadharshini Sivakumaran, Mark M. W. Chong, Xin Liu, Tara Karnezis, Nadeeka Bandara, Kaloyan Takov, Cameron J. Nowell, Stephen Wilcox, Mitch Shambrook, Andrew F. Hill, Nicole C. Harris, Andrew E. Newcomb, Padraig Strappe, Ramin Shayan, Damián Hernández, Jordan Clarke, Eric Hanssen, Sean M. Davidson, Gregory J. Dusting, Alice Pébay, Joshua W. K. Ho, Nicholas Williamson, Shiang Y. Lim

**Affiliations:** 1 The Bio21 Molecular Science and Biotechnology Institute, University of Melbourne, Victoria, Australia; 2 Victor Chang Cardiac Research Institute, New South Wales, Australia; 3 St Vincent’s Clinical School, University of New South Wales Sydney, New South Wales, Australia; 4 St Vincent’s Institute of Medical Research, Victoria, Australia; 5 Departments of Medicine and Surgery, University of Melbourne, Melbourne East, Victoria Australia; 6 School of Biomedical Sciences, Charles Sturt University, New South Wales, Australia; 7 The Hatter Cardiovascular Institute, University College London, London, UK; 8 Drug Discovery Biology, Monash Institute of Pharmaceutical Sciences and Department of Pharmacology, Monash University, Victoria, Australia; 9 Walter and Eliza Hall Institute of Medical Research, Victoria, Australia; 10 Department of Biochemistry and Genetics, La Trobe Institute for Molecular Science, La Trobe University, Bundoora, Victoria Australia; 11 Department of Cardiothoracic Surgery, St. Vincent’s Hospital Melbourne, Victoria, Australia; 12 School of Health, Medicine and Applied Sciences, Central Queensland University, Queensland, Australia; 13 Centre for Eye Research Australia, Royal Victorian Eye and Ear Hospital Melbourne East, Victoria, Australia

## Abstract

The benefits of adult stem cells for repair of the heart have been attributed to the repertoire of salutary paracrine activities they appear to exert. We previously isolated human W8B2^+^ cardiac stem cells (CSCs) and found they powerfully influence cardiomyocytes and endothelial cells to collectively promote cardiac repair and regeneration. Here, the complexity of the W8B2^+^ CSC secretomes was characterised and examined in more detail. Using ion exchange chromatography to separate soluble proteins based on their net surface charge, the secreted factors responsible for the pro-survival activity of W8B2^+^ CSCs were found within the low and medium cation fractions. In addition to the soluble proteins, extracellular vesicles generated from W8B2^+^ CSCs not only exhibited pro-survival and pro-angiogenic activities, but also promoted proliferation of neonatal cardiomyocytes. These extracellular vesicles contain a cargo of proteins, mRNA and primary microRNA precursors that are enriched in exosomes and are capable of modulating collectively many of the cellular pathways involved in protein metabolism, cell growth, as well as cellular responses to stress and organisation of the extracellular matrix. Thus the W8B2^+^ CSC secretome contains a multitude of bioactive paracrine factors we have now characterised, that might well be harnessed for therapeutic application for cardiac repair and regeneration.

## Introduction

The increasing prevalence and high mortality of heart disease demands a continued search for innovative approaches to management that might restore cardiac function. Unlike some other organs, the heart lacks the intrinsic ability to adequately repair itself^1^. Stem cell-based therapies to repair and regenerate injured myocardium represent new avenues to address this unmet medical need. Although trials of such therapies have in general been encouraging, meta-analysis reveals they have achieved mixed outcomes to date^2,3^.

In damaged hearts, the alleged ability of adult stem cells to differentiate to functional cardiomyocytes *in vivo*, to directly regenerate new muscle, has been a controversial. We and others have shown that adult stem cells transplanted into infarcted myocardium rarely differentiate into cardiomyocytes and cannot account for the observed cardioreparative effect^4,5^. The hostile microenvironment of infarcted myocardium lacks the supportive niche for the engraftment and survival of transplanted cells, limiting their regenerative potential via differentiation into cardiovascular cells. Furthermore, this direct mechanism will only work if transplanted cells can engraft functionally and be maintained in the host myocardium for long periods whilst simultaneously becoming electrically synchronous with the surrounding cardiomyocytes to avoid potential arrhythmic complications^6,7^. In fact, transplantation of stem cells has been supposed to produce a therapeutic benefit mainly through release of a complex suite of trophic paracrine factors in the secretome, that indirectly promote endogenous repair. Supporting the significance of such paracrine mechanisms are experimental studies demonstrating that the administration of conditioned media from stem cells were able to confer cardioreparative effects without requiring the physical transplantation of stem cells within the infarcted heart^8,9^. The secretomes of stem cells have been shown to enrich the microenvironment of infarcted myocardium – these acting by various mechanisms including improving cell survival, enhancing angiogenesis, regulating inflammation, reducing adverse remodelling, and recruiting and activating endogenous resident stem cells for cardiac repair^8,9^. Besides being released as soluble factors, these paracrine factors can also be released in extracellular vesicles (EVs) such as microvesicles and exosomes^5,10^. Of particular interest are the exosomes (50–150 nm in diameter), which carry a range of bioactive factors such as cytokines, growth factors, lipids, mRNAs and microRNAs. Moreover, exosomes can transfer these multiple factors to other cells by fusing with the plasma membrane and disgorging their contents therein^11,12^. Exosomes isolated from human stem cells have been shown to protect the infarcted myocardium^11,13,14^, raising the possibility that the EVs and exosomes might be exploited as alternative, non-cellular therapeutic approaches for cardiac repair and regeneration.

W8B2 is a specific marker for the isolation of bone marrow-derived stem cells with high proliferative potential (W8B2^+^CD271^bright^ population)^15^. Using this unique surface marker, we have previously isolated a novel population of W8B2^+^ cardiac stem cells (CSCs) from human atrial appendages^4^. These are distinct from other stem cell populations previously identified in the heart including C-KIT^+^, SCA-1^+^ and ISL-1^+^ cardiac stem cells^4^. Intramyocardial transplantation of human W8B2^+^ CSCs into immunocompromised rats one week post myocardial infarction improved cardiac function and reduced maladaptive remodelling of the left ventricle for at least 3 weeks post-treatment^4^. These beneficial effects can likely be attributed to the paracrine action of W8B2^+^ CSCs for there is no evidence that the transplanted cells engraft successfully nor differentiate into cardiovascular cell types in this setting^4^. Interestingly, conditioned medium harvested from W8B2^+^ CSCs displayed a range of biological activities including pro-survival, pro-angiogenic, and pro-migratory effects on endothelial cells and neonatal rat cardiomyocytes, clearly superior to those exerted by W8B2^-^ cells, CKIT^+^ CSCs and bone marrow-derived stem cells^4^. However, the individual factors that were responsible for these cytoprotective and regenerative effects remained unknown. Here, we employ ion exchange chromatography and ultracentrifugation to separate charged proteins and extracellular vesicles in the conditioned media, respectively. We subsequently applied fractions in several *in vitro* bioassays that reflect cardiac repair and regeneration capacity (angiogenesis, cell survival and cardiomyocyte proliferation) in order to profile the biological activities of the separated proteins and extracellular vesicles. Finally, we used proteomic and transcriptomic approaches to characterise and profile the secretome constituents of these unique W8B2^+^ CSCs.

## Results

### Effect of soluble proteins secreted by W8B2^+^ CSCs on cell survival, angiogenesis and cardiomyocyte proliferation

**Figure 1:**
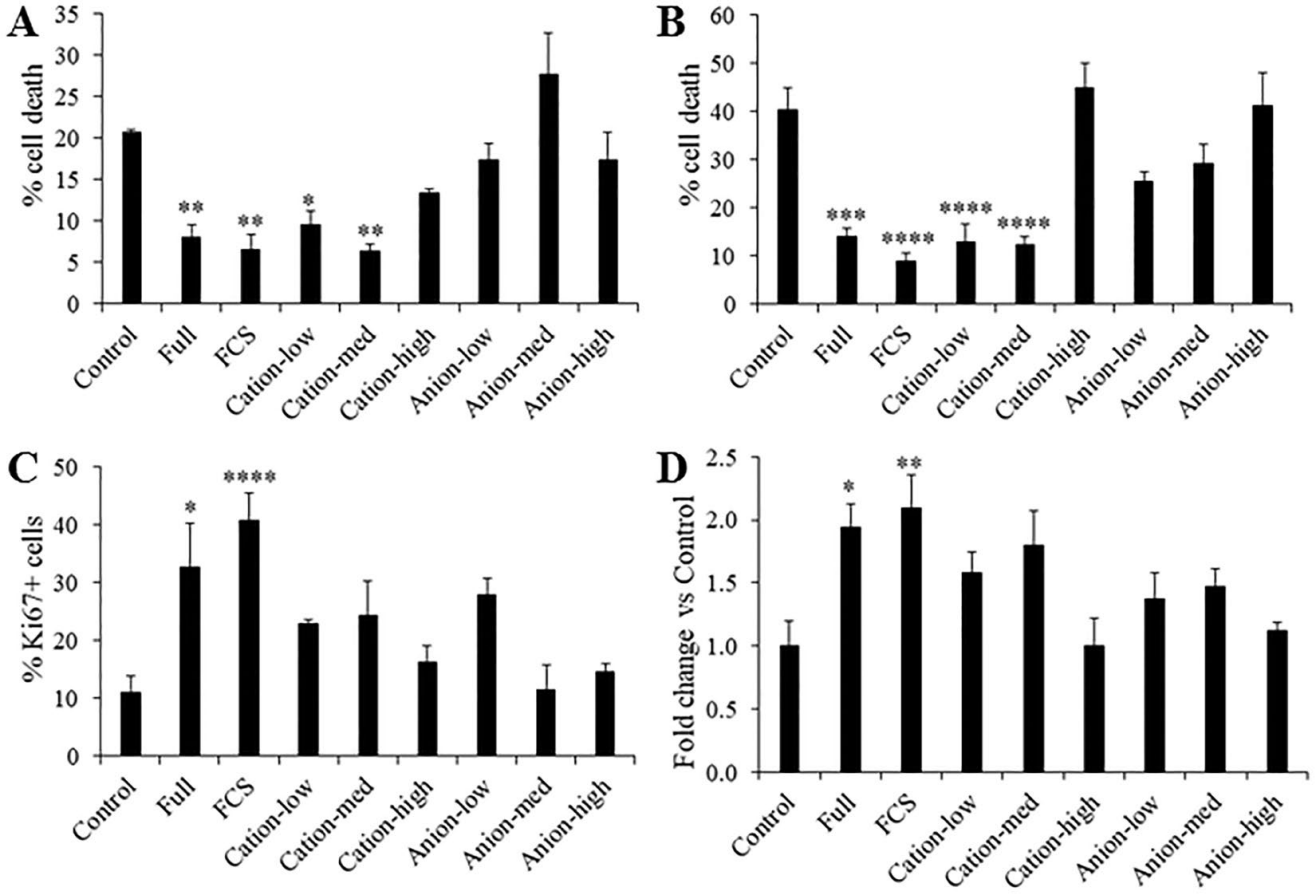
Biological activity profile of W8B2^+^ CSC-conditioned medium fractionated by ion exchange chromatography. The effect of conditioned medium on survival of (**A**) neonatal rat cardiomyocytes and (**B**) human cardiac microvascular endothelial cells. (**C**) Proliferation of neonatal rat cardiomyocytes represented by percentage of Ki67^+^ cells. (**D**) Pro-angiogenic tube formation of human cardiac microvascular endothelial cells, assessed by the number of loops formed *in vitro*. Serum-free medium and serum-free medium supplemented with 5% FCS (FCS) were served as Control and as a positive control (FCS), respectively. Data are shown as mean ± SEM from 4–6 independent experiments. *p < 0.05; **p < 0.01; ***p < 0.001; and ****p < 0.0001 versus Control by one-way ANOVA. Control, serum-free medium; Full, unfractionated conditioned medium; FCS, serum-free medium supplemented with 5% FCS.

W8B2^+^ CSCs cultured in serum-free medium under normoxic (20% O_2_) condition for 3 days have normal spindle-shaped, fibroblastic morphology and trypan blue exclusion assay indicated 92.1 ± 1.6% of viable cells (n = 8). To determine whether the soluble proteins secreted by W8B2^+^ CSCs promote survival of the main cell types in the heart, cardiomyocytes (neonatal rat cardiomyocytes) and endothelial cells (human cardiac microvascular endothelial cells, HCMECs) were subjected to hypoxia and serum deprivation, which simulates the *in vivo* ischaemic condition. In neonatal rat cardiomyocytes subjected to simulated ischaemia, the presence of unfractionated conditioned medium significantly reduced cell death from 20.7 ± 0.5% in control to 8.0 ± 1.7% (p < 0.01, n = 4). This pro-survival effect was comparable to that observed in a positive control (5% fetal calf serum, FCS). The pro-survival effect of conditioned medium was retained in the low cation and medium cation fractions of W8B2^+^ CSC conditioned medium, but not in the high cation fraction or the anion fractions (Fig. 1A). Similar results were obtained with HCMECs subjected to simulated ischaemia that treatment with 5% FCS, unfractionated, low cation and medium cation fractions of W8B2^+^ CSC conditioned medium significantly reduced cell death when compared to control group (Fig. 1B). The cytoprotective effect of 5% FCS, unfractionated, low cation and medium cation fractions of W8B2^+^ CSC conditioned medium were comparable among groups and did not differ statistically (Fig. 1A,B). Neonatal rat cardiomyocytes exhibit a low level of basal proliferative activity. Compared with the control group, treatment with unfractionated conditioned medium for 24 hours significantly increased the number of proliferative cardiomyocytes (Ki67^+^cTnT^+^ cells), to a comparable level to that observed with a positive control (5% FCS) (Fig. 1C). However, the cation and anion fractions of W8B2^+^ CSC conditioned medium failed to stimulate the proliferation of neonatal rat cardiomyocytes (Fig. 1C). To investigate the pro-angiogenic paracrine effect of W8B2^+^ CSCs, a 2-dimensional Matrigel endothelial network assay was employed. Compared to the control group, unfractionated conditioned medium and 5% FCS (as positive control) were able to stimulate HCMECs to form a capillary-like network on Matrigel. This significant improvement was observed in term of number of complete ring formed (Fig. 1D), but not the total tubule length, branch points, number of segments, average tubule thickness and connected set (Supplementary Fig. S1). However, the pro-angiogenic effect of W8B2^+^ CSC conditioned medium was not observed in the cation or anion fractions of W8B2^+^ CSC conditioned medium (Fig. 1D). Our data thus demonstrates that the low and medium cation fractions of W8B2^+^ CSC conditioned medium contain active components that promote cell survival.

### Proteomic profiling of protein expression in W8B2^+^ CSC-conditioned medium

**Figure 2:**
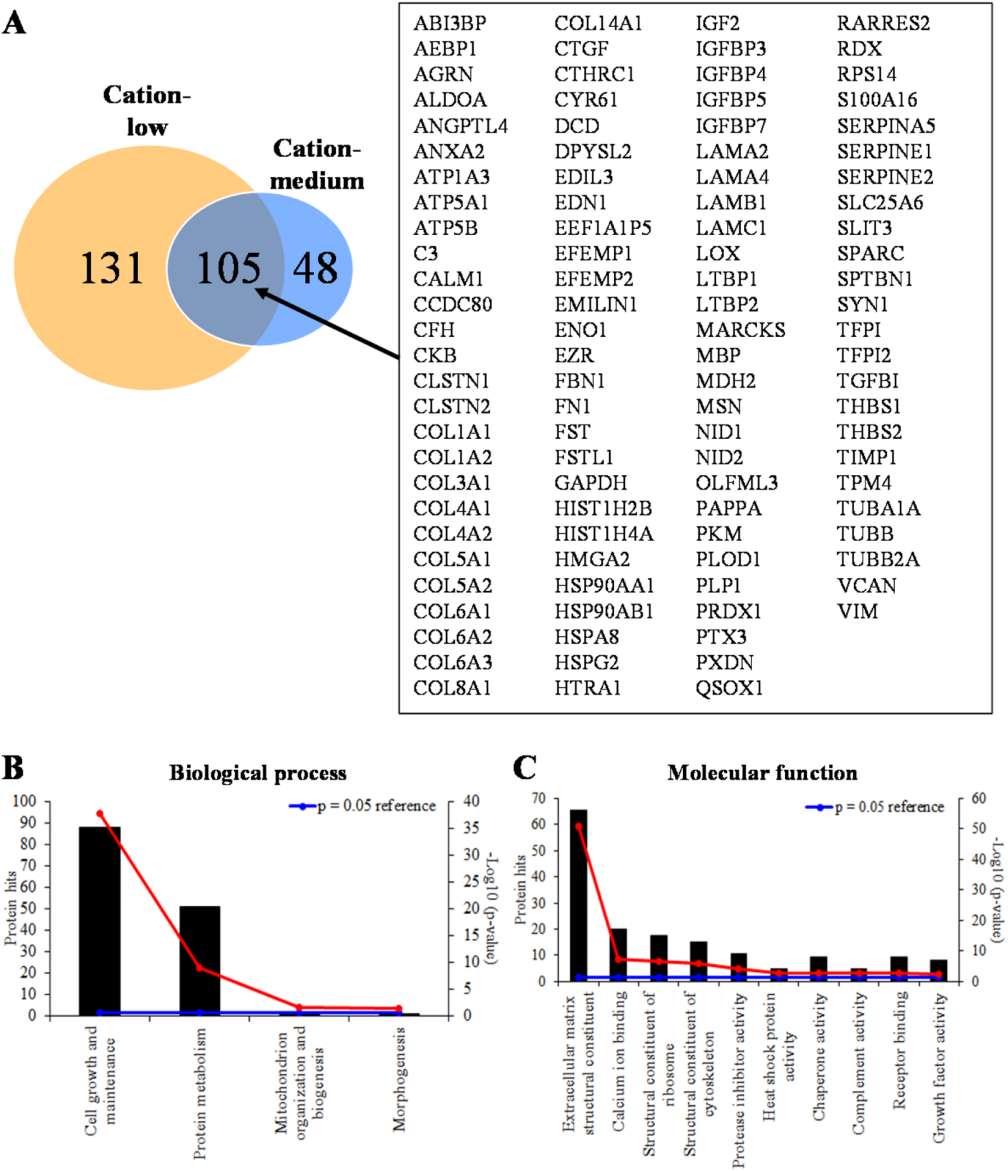
Functional analysis of proteins identified from W8B2^+^ CSC conditioned medium. (**A**) Venn diagram showing a total of 284 proteins in the combined low and medium cation fractions. (**B**) Biological processes and (**C**) molecular functions of these 284 proteins analysed by FunRich functional enrichment analysis (hypergeometric test p-value is indicated by red line and p < 0.05 means significant enrichment).

We next performed mass spectrometry analyses of these fractions to reveal the protein expression. The unfractionated conditioned medium contains 648 proteins (Supplemental Data set 1). A total of 236 proteins were identified in the low cation fraction (Fig. 2A and Supplementary Data Set 2). Of these identified proteins, 105 were also found in the medium cation fraction (Fig. 2A and Supplementary Data Set 2). FunRich analysis^16^ showed that the three enriched biological processes regulated by these proteins are cell growth and maintenance, protein metabolism and mitochondrion organization and biogenesis (Fig. 2B). The enriched molecular functions of the identified proteins included extracellular matrix structural constituent, calcium ion binding, ribosome and cytoskeleton structural constituent, protease inhibitor activity, heat shock protein activity, chaperon activity, complement activity, receptor binding and growth factor activity (Fig. 2C).

### Effects of extracellular vesicles secreted by W8B2^+^ CSCs on cell survival, angiogenesis and cardiomyocyte proliferation

**Figure 3:**
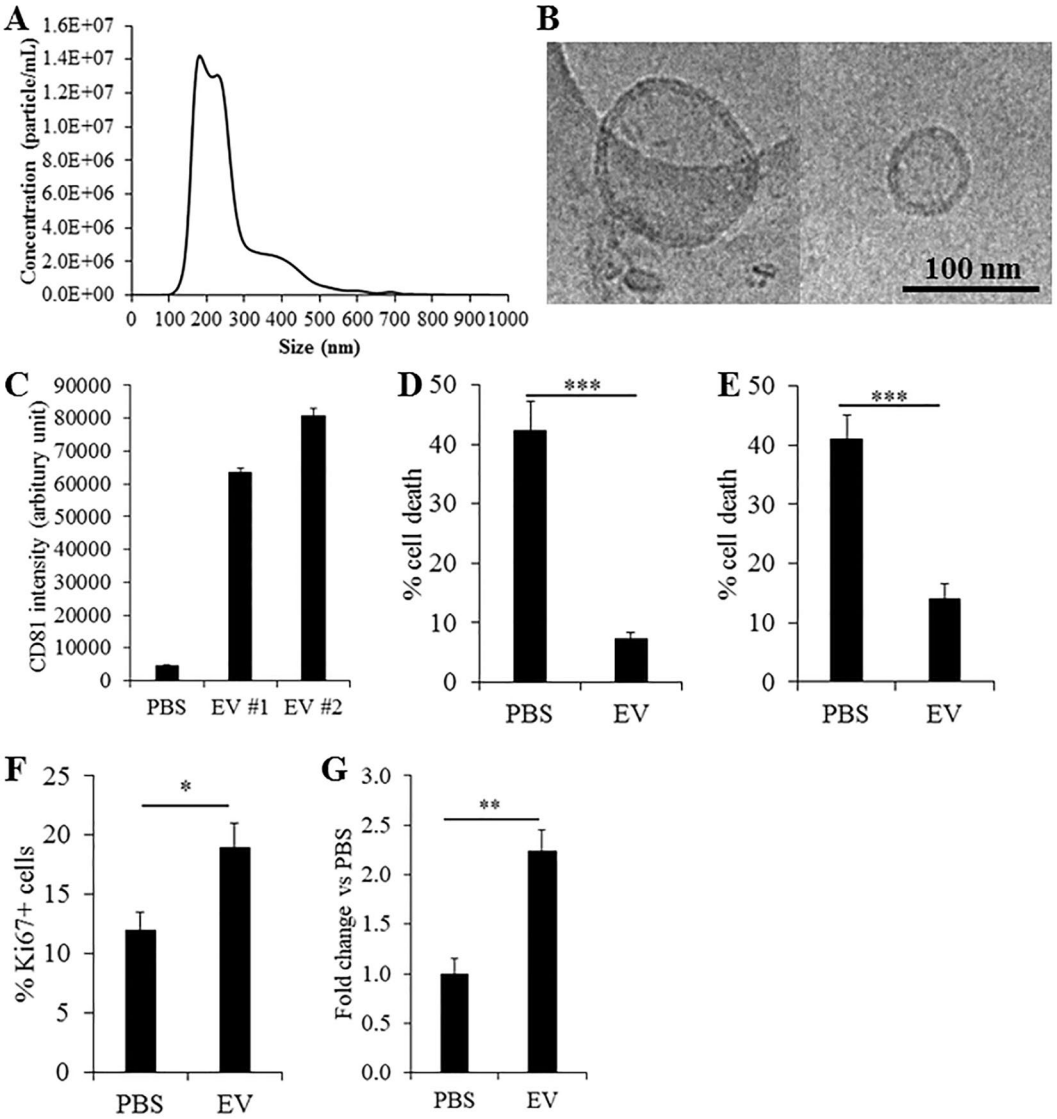
Molecular and biological regenerative properties of extracellular vesicle fractions of W8B2^+^ CSC-conditioned medium. (**A**) Particle size distribution shows extracellular vesicles with an average modal size of 201 ± 12 nm. (**B**) Electron micrograph images of extracellular vesicles shows near-spherical shape of double membraned vesicles. (**C**) DELFIA protein analysis shows the expression of CD81 in the extracellular vesicles isolated from 2 different biological samples. The effect of extracellular vesicles on survival of (**D**) neonatal rat cardiomyocytes and (**E**) human cardiac microvascular endothelial cells in culture after simulated ischaemia. The effect of extracellular vesicles on (**F**) proliferation of neonatal rat cardiomyocytes proliferation and (**G**) pro-angiogenic tube formation of human cardiac microvascular endothelial cells, assessed by the number of loops formed. Data are shown as mean ± SEM from 4–6 independent experiments. *p < 0.05; **p < 0.01; ***p < 0.001 versus PBS by Student’s *t* test. PBS, phosphate-buffered saline; EV, extracellular vesicles.

Serial differential centrifugation and ultracentrifugation of W8B2^+^ CSC-conditioned medium resulted in an EV pellet containing both exosomes and microvesicles with an average modal size of 201 ± 12 nm (Fig. 3A). Nanoparticle tracking analysis showed a high number of EVs secreted by W8B2^+^ CSCs with approximately 2 × 10^9^ particles released by 10^6^ cells after 72 hours. Qualitative assessment with cryogenic transmission electron microscopy showed that the exosomes in the EV fraction have the expected spherical shape with bilayer membranes (Fig. 3B). DELFIA analysis confirmed the expression of the exosome canonical marker CD81 in the EV/exosome fraction of W8B2^+^ CSC conditioned medium (Fig. 3C). *In vitro* bioassays were then employed to experimentally assess the functional properties of EVs as biologically active mediators of the W8B2^+^ CSC paracrine effect. In the cell survival assay, treatment with W8B2^+^ CSC-derived EVs effectively protected neonatal rat cardiomyocytes (7.4 ± 0.9% cell death vs. 42.4 ± 4.8% in PBS vehicle control, p < 0.001, n = 4; Fig. 3D) and HCMEC (14.0 ± 2.6% cell death vs. 41.1 ± 4.0% in PBS vehicle control, p < 0.001, n = 4; Fig. 3E) from simulated ischaemia-induced cell death. Treatment with W8B2^+^ CSC-derived EV for 2 days significantly increased the number of Ki67^+^ proliferating neonatal rat cardiomyocytes (18.9 ± 2.0% vs. 11.9 ± 1.6% in PBS control, p < 0.05, n = 4; Fig. 3F). In the Matrigel endothelial cell tube formation assay, treatment with W8B2^+^ CSC-derived EVs significantly increased the number of complete ring formed (Fig. 3G), but not the total tubule length, branch points, number of segments, average tubule thickness and connected set (Supplementary Fig. S2).

### Analysis of the cargo of extracellular vesicles secreted by W8B2^+^ CSCs

**Figure 4:**
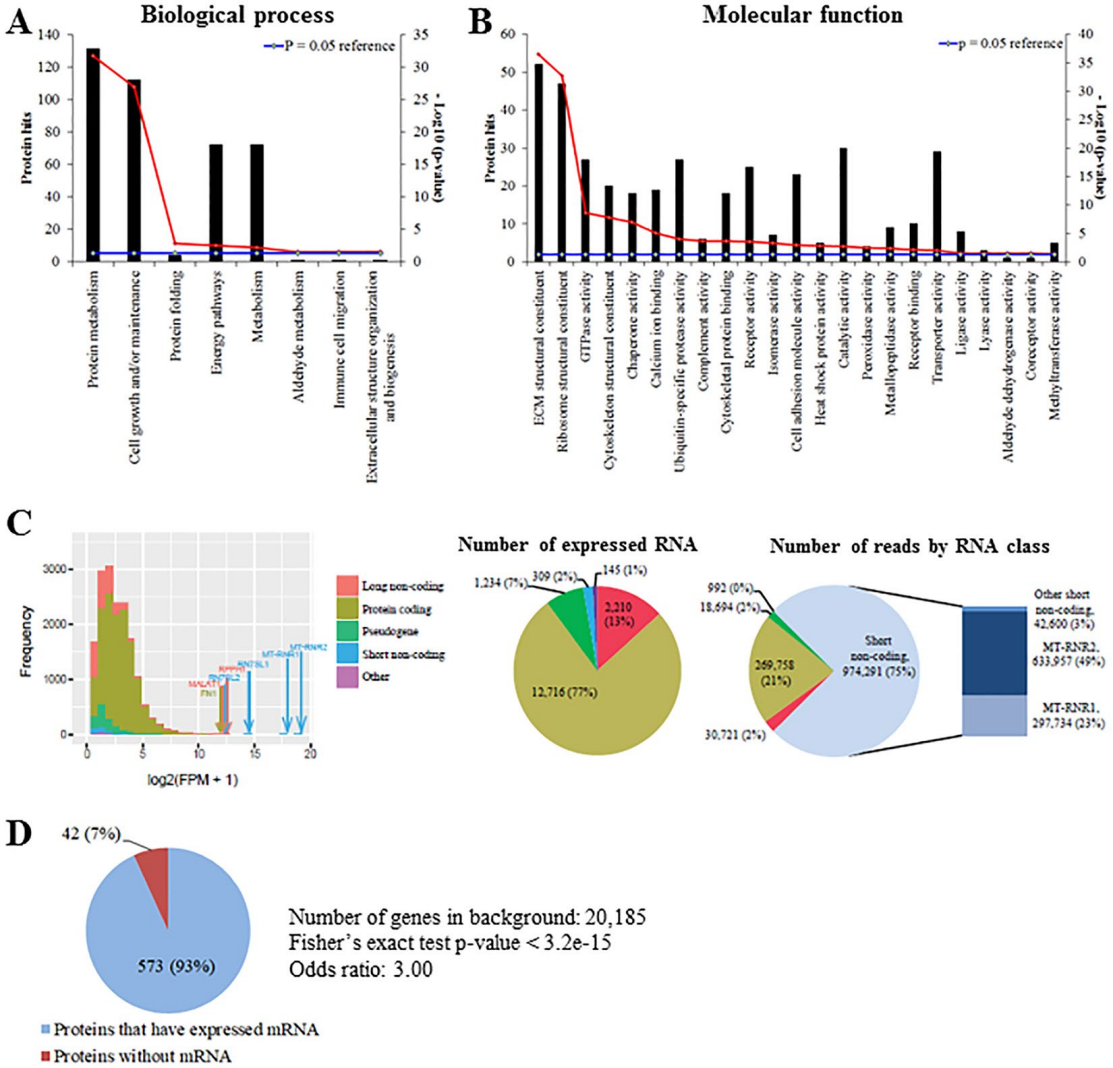
Proteomic and RNA sequencing analysis of extracellular vesicles derived from W8B2^+^ CSCs. (**A**) Biological processes and (**B**) molecular functions of proteins identified in extracellular vesicles and analysed by FunRich (hypergeomic test p-value is indicated by red line and p < 0.05 means significant enrichment). ECM, extracellular matrix. (**C**) Left: The expression levels, in terms of log_2_ transformed sequence Fragment Per Million (FPM), of five classes of transcripts. Right: The number of expressed transcripts and number of reads that are aligned to each of the five transcript classes. (**D**) A high proportion of the 615 proteins identified by mass spectrometry have overlapped expressed mRNA in the extracellular vesicles.

The proteomic content of EVs secreted by W8B2^+^ CSCs was characterised by mass spectrometry analysis and a total of 602 proteins were recorded with fibronectin being the most abundant protein (Supplementary Data Set 3). Many of these proteins can interact with each other to execute various biological pathways. The protein-protein interaction network also reveal several hub nodes including ITGA2, ITGB1, HSP90AB1, APP, THBS1, FN1, COL1A1, YMHAZ and YMHAG (Supplementary Fig. S3). Proteins identified include those typically expressed by exosomes (~74%), including PDCD6IP (Alix), CD9, CD81, Tsg101, HSPA1A (Hsp70) and Rab5, which were absent in the low and medium cation fractions of W8B2^+^ CSC conditioned medium. These proteins were appropriately processed by FunRich and were found to associate with 8 biological processes at p < 0.05 based on hygergeometric test (Fig. 4A). These processes are; protein metabolism, cell growth and maintenance, protein folding, energy pathway, metabolism, aldehyde metabolism, immune cell migration, and extracellular structure organisation and biogenesis (Fig. 4A). On a molecular function basis, proteins functioning as extracellular matrix and ribosome structural constituents were enriched in EVs of W8B2^+^ CSCs (Fig. 4B).

RNA sequencing was performed to characterise the total RNA content of EVs secreted by W8B2^+^ CSCs (Supplementary Data Set 4). Among ~1.3 million pair-end RNA sequencing reads that are uniquely aligned to the human genome, 75% of the reads mapped to small non-coding RNA, mostly consisting of the two mitochondrial ribosomal RNA (rRNA) genes, MT-RNR1 and MT-RNR2 (Fig. 4C). Protein-coding mRNA accounts for 21% of the reads, distributed over 12,716 genes, which accounts for 77% of the 16,614 expressed transcripts found in the EVs (Fig. 4C). There were also a small proportion of reads mapping to long non-coding transcripts (2.4% of the reads over 2,210 transcripts) and pseudogenes (1.5% of reads over 1,234 transcripts). Among the 615 proteins identified in the proteomic screen (Supplementary Data Set 3), 573 (93%) of them have their corresponding transcripts expressed in the EVs and were captured by RNA sequencing (Fig. 4D). Enrichment analysis of all identified proteins and RNA showed that EV proteins that have expressed mRNA were enriched for cellular junctions and extracellular matrix organization as well as some immune related GO terms and pathways (Supplementary Fig. S4). Since microRNAs (miRNAs) are known to be important functional constituents of the EV/exosome RNA cargo, the libraries were also analysed for miRNA content. Altogether, 20 high confidence expressed miRNA were identified and the presence of miR1244 and miR3648/3687 precursors in the EVs was further validated by PCR (Supplementary Fig. S5). It is conceivable that these miRNA precursors are processed into mature miRNAs in the targets, and could therefore have gene regulatory impacts. We therefore also performed target gene prediction using TargetScan^17^ (see Methods for details). Gene set analysis showed that these miRNA target genes were significantly enriched (adjusted p value < 0.05) for GO terms associated with apoptosis, angiogenesis and cell proliferation (Table 1).

**Table 1:** List of key gene sets enriched by the predicted miRNA gene targets of the exosome miRNA.

**microRNA**	**Gene set**	**FDR**	**Term ID**	**Predicted target genes that overlap with this gene set**
miR-3615	Positive regulation of execution phase of apoptosis	0.00247	GO:1900119	BAX, TP53
miR-4479	Regulation of Insulin-like Growth Factor (IGF) transport and uptake by Insulin-like Growth Factor Binding Proteins (IGFBPs)	0.0079	REAC:381426	VGF, APOE, MFI2, VWA1, GAS6
miR-3615	Regulation of execution phase of apoptosis	0.00899	GO:1900117	BAX, TP53
miR-3687	Negative regulation of smooth muscle cell proliferation	0.0103	GO:0048662	HMOX1, OGN
miR-1244	Positive regulation of cell cycle G1/S phase transition	0.0165	GO:1902808	FAM83D, PPP1R1C
miR-6087	Positive regulation of execution phase of apoptosis	0.0183	GO:1900119	BAX, PTGIS
miR-3615	Positive regulation of cell cycle process	0.019	GO:0090068	BAX, TP53, PRDM9, PRKCE, INS
miR-1244	Angiogenesis	0.0195	GO:0001525	RHOA, SAT1, ANXA3, VEGFC, ANGPT1, NOS3, TDGF1
miR-1244	Cell cycle G1/S phase transition	0.0203	GO:0044843	FAM83D, PLAGL1, POLE3, PPP1R1C, CCNE2, TRIM71
miR-1244	Sprouting angiogenesis	0.0246	GO:0002040	RHOA, ANGPT1, TDGF1
miR-6087	Execution phase of apoptosis	0.0257	GO:0097194	BAX, BNIP1, PTGIS, CIDEB, DFFB
miR-1244	Cell migration involved in sprouting angiogenesis	0.0376	GO:0002042	RHOA, TDGF1
miR-6087	Regulation of cardiac muscle cell proliferation	0.0448	GO:0060043	TP73, NOG

FDR: False Discovery Rate, calculated based on Benjamini Hochberg-corrected p values (Fisher’s exact test).

## Discussion

This study clearly shows that the secretome of human W8B2^+^ CSC contains biological factors that are capable of promoting cell survival, angiogenesis and proliferation of cardiomyocytes. These findings support our previous supposition that the cardioreparative effect of W8B2^+^ CSC^4^ *in vivo* is likely attributable to release of potent cardioreparative paracrine factors. To focus on the chemical characteristics of these paracrine factors responsible for the beneficial effects of the conditioned medium, microscale separation of the unfractionated conditioned medium was performed using ion exchange chromatography. We observed that the pro-survival factors reside within the low and medium cation fractions of conditioned medium: this contained 284 proteins that appear to have roles in cell maintenance and growth, protein metabolism and mitochondrial biogenesis. Moreover, the pro-survival factors are likely components of the 105 proteins common between the low and medium cation fractions, and these include insulin-like growth factor binding protein-3 (IGFBP-3), IGFBP-7, insulin-like growth factor-2, follistatin and SERPINE1. In particular, IGFBP-3 has been shown to promote endothelial cell survival under conditions of serum-starvation^18^. Note that the degree of cardiomyocyte death in the control group containing concentrated serum-free basal medium (Fig. 1A) was less than the PBS vehicle control (Fig. 3D), probably due to the presence of pleiotropic antioxidants such as ascorbic acid^19^, α-tocopherol^19^, nicotinic acid^20^ and hypoxanthine^21^ in the basal medium for all are cytoprotective at high concentration. Interestingly, the paracrine actions of W8B2^+^ CSC conditioned medium in promoting angiogenesis and cardiomyocyte proliferation were not found in cation or anion fractions of conditioned medium. These findings may suggest that factors responsible for the pro-mitotic and pro-angiogenic effects of W8B2^+^ CSC secretome may be in the neutral fraction or attributed to a combination of multiple factors from different fractions, which requires further investigation.

In adult stem cells, EVs and exosomes play key roles in autocrine and paracrine signalling^22^. Using the ultracentrifugation method, we isolated the EV fraction of W8B2^+^ CSC secretomes that met the criteria recommended by the International Society For Extracellular Vesicles^23^. Consistent with other studies on stem cell-derived EVs, those secreted by W8B2^+^ CSCs are heterogeneous in size and contain nano-sized double membranous exosomes enriched with exosome-associated proteins and RNA, including Alix, Tsg101, HSP70 and tetraspanins (CD9 and CD81)^23^. The average modal size of EVs found in the present study by nanoparticle tracking analysis was somewhat larger (~200 nm) than the typical size distribution of exosomes (50–150 nm), possibly due to different methods used to measure the size of EVs. Nanoparticle tracking analysis (Fig. 3A) estimates EV hydrodynamic sizes, which are usually larger than their innate geometric sizes as characterised by cryogenic transmission electron microscopy (Fig. 3B)^24^. Other factors contributing to the size variability include different storage conditions (freshly isolated versus overnight storage at 4 °C), isolation techniques and the cell types involved^24^. Importantly, the EV fraction of W8B2^+^ CSC secretome exhibits all of pro-survival, pro-mitotic and pro-angiogenic properties *in vitro* that reflect and could be responsible for cardiac repair and regeneration (Fig. 3). This concurs with other studies where EVs and exosomes isolated from other cardiac stem cell types, such as CKIT^+^ CSCs^25^ and cardiosphere-derived cells^26,27^ have been reported to exert cardioprotective effects *in vitro* and *in vivo*. The cardioprotective effect of CSC-derived exosomes has been largely attributed to their miRNA contents such as miR-146a, miR-210 and miR-132^26,27^. Indeed, EVs of W8B2^+^ CSCs were found enriched with miRNA precursors and miRNAs known to regulate cell survival (miR-3615 and miR-6087), angiogenesis (miR-1244) and cell proliferation (miR-3687, miR-1244, miR-3615 and miR-6087), and these could be further processed into mature miRNAs in the recipient cells and responsible for the biological activities of EVs reported here (Fig. 3D–G). Future studies should confirm the presence of these mature miRNAs as well as using miRNA inhibitors to confirm the involvement of these miRNAs, and miRNA mimics could be tested to recapitulate the effects of the EVs studied here. However, it may be that a combination of several miRNAs as provided by these EVs is required in order to confer the benefits observed.

Besides miRNAs, our RNA sequencing analysis also demonstrated that the EVs secreted by W8B2^+^ CSCs contain a few, very highly expressed, small non-coding RNA, including mitochondrial rRNA, MT-RNR1 and MT-RNR2 (Supplementary Fig. S6 for their genome browser view and their validation by RT-PCR). A previous study profiling the extracellular RNA repertoires of human carcinoma cell lines has also reported the abundance of these two mitochondrial rRNA species in the secreted EVs^28^. Although the presence of mitochondrial DNAs and RNAs has been noted in the EVs released by various cell types including rat astrocytes, human endothelial cells and other human cell lines^28–31^, their functions, if any, as part of the cargo of EVs remains poorly understood. Their inclusion into the EV repertoire could also be due to the crosstalk between autophagy or mitophagy and exosome biogenesis, for these processes share similar intracellular pathways in endosome formation^32,33^. Contamination by mitochondria is unlikely because any mitochondria in the conditioned medium should have been pelleted and removed during the 10,000 *g* centrifugation step of EVs isolation^34^. Furthermore, mitochondrial ultrastructure was not detected in the electron microscopy analysis of the EV fractions, and no mitochondrial-specific proteins were detected in the proteomic analysis. Long non-coding RNAs possess various regulatory functions at epigenetic, transcriptional and post-transcriptional levels^35^. Long non-coding RNAs such as MALAT1 (metastasis-associated lung adenocarcinoma transcript 1), NEAT1 (nuclear enriched abundant transcript 1), GAS5 and RMRP have previously been shown expressed in EVs of various human cell types in response to cell stress^36,37^ and were found to be highly expressed in the EVs of human W8B2^+^ CSCs. MALAT1 is one of the most established and conserved long non-coding RNAs frequently found in exosomes of human cells and plays a role in regulating cellular proliferation and angiogenesis, and has been proposed as a biomarker for the diagnosis of cancer metastasis^38–40^. Induction of NEAT1 has been found to promote survival and proliferation of cancer cells^41^. RMPR is a mitochondrial RNA-processing endoribonuclease^42^, which has previously been found to associate with the HeLa and C33A exosomes and can affect cell viability upon exosomal entry into target cells^37^. Future studies should further validate these long non-coding RNAs in the EVs secreted by W8B2^+^ CSCs. The complexity and abundance of this extracellular RNA repertoire in W8B2^+^ CSC EVs may well be responsible for their pleiotropic actions through transcriptional and translational regulations of gene expression in target cells.

A significant number of proteins identified in the proteomic analysis (93%, Fig. 4D) also have their corresponding mRNA captured in the EVs of W8B2^+^ CSCs. This proteogenomic analysis, together with the abundance of ribosomes and rRNAs found within the EVs, could suggest that proteins were incorporated into EVs during the process of their translation. In fact, the top 5 biological pathways identified by FunRich are crucial players in mRNA translation (Supplemental Dataset 2). These pathways include metabolism of mRNA, eukaryotic translation elongation, metabolism of RNA, peptide chain elongation and termination of eukaryotic translation. Importantly, FunRich biological pathway analysis also reveals that EVs of W8B2^+^ CSCs are enriched with proteins implicated in cell survival (signalling pathways for PI3K-Akt, epidermal growth factor, nerve growth factor, hepatocyte growth factor and HIF-1α transcription factor network). In addition, crucial regenerative biological functions are represented by factors involved in angiogenesis (VEGF and VEGFR signalling network, endothelin-mediated signalling pathway, eNOS regulated pathway) and cell proliferation (insulin-like growth factor-mediated signalling pathway, cell cycle and mitotic regulation, Notch signalling pathway, Ephrin-B signalling pathway). The breadth of proteins, mRNA and miRNA found in the secretome of human W8B2^+^ CSCs suggests the likelihood that secreted factors synergise in concert, rather than acting individually, to drive the observed repair and regenerative outcomes, although clearly this remains to be proven. Thus further studies are warranted to elucidate individually the key bioactive factors that endow the cardioreparative effect of W8B2^+^ CSCs.

In summary, the secretome of W8B2^+^ CSCs exhibits pro-survival, pro-angiogenic and pro-mitogenic effects on neonatal rat cardiomyocytes and human cardiac microvascular endothelial cells that collectively support roles for key proteins, mRNAs and small RNAs as biological mediators of cardiac repair. We have provided a novel, comprehensive, initial characterisation of the proteomic and genomic content of W8B2^+^ CSC secretome that shows abundant cytoprotective and regenerative factors (i.e. low cation, medium cation and EV fractions), thus providing a valuable resource for future studies of this secretome. This includes a direct comparison between the soluble fractions and EVs to determine whether EVs are the main paracrine mediators of W8B2^+^ CSCs, as well as further microscale protein separation with either ion exchange chromatography or gel-filtration chromatography. Future studies are also required to ascertain the cardiac repair and regenerative effects of the W8B2^+^ CSC secretome in animal models of myocardial infarction. From a clinical perspective, harnessing this cardioreparative secretome of W8B2^+^ CSCs for cardiovascular therapy might offer the opportunity to achieve the regenerative benefit of stem cell therapies in a more controlled, reversible and safe manner than is offer by traditional stem cell transplantation. This could allow the use of allogeneic sources of cells as scalable, off-the-shelf formulations. Furthermore, optimal cardiac tissue could be sourced to maximise the potency and to avoid comorbidities such as diabetes, allowing appropriate quality assurance and consistency of end-products for therapeutic application.

## Methods

### W8B2^+^ cardiac stem cells

W8B2^+^ CSCs were isolated from atrial appendages as previously described^4^. Human tissues were collected with informed consent according to the Australian National Health and Medical Research Council guidelines and with approval from the Human Research Ethics Committee-A of St. Vincent’s Hospital (HREC-A 07/08). All experiments were performed in accordance with relevant guidelines and regulations of Australian National Health and Medical Research Council.

### Isolation of neonatal rat cardiomyocytes

Neonatal rat cardiomyocytes were isolated from the ventricles of 1–3 days old Sprague-Dawley rats according to the manufacture’s procedure (Neonatal Cardiomyocyte Isolation System, Worthington Biochemical, NJ, USA). The experimental procedures were approved by the Animal Ethics Committee of St Vincent’s Hospital Melbourne (AEC 001/13; Victoria, Australia) and were conducted accordance with the Australian National Health and Medical Research Council guidelines.

### Collection of conditioned media

W8B2^+^ CSCs (passage 2–4) were cultured to 95% confluence in T75 culture flasks, washed twice with PBS, and replenished with 10 mL of serum-free basal medium (25% endothelial basal medium and 75% M199 medium) for 72 hours at 37 °C in a humidified incubator containing 5% CO_2_. Collected media were filtered through a 0.2 µm filter, concentrated by a factor of 50 times (final volume of 200 µL) and desalted using Amicon Ultra-15 centrifugal filter devices with 3000 molecular weight cut off membrane (Millipore, MA, USA).

### Ion exchange chromatography

Charged proteins in the conditioned medium harvested from W8B2^+^ CSCs were separated and purified by cation and anion exchange chromatography (HiTrap SP and Q, 1 mL column, GE Healthcare BioSciences, Sweden) using BioLogic LP low-pressure chromatography system (Bio-Rad, CA, USA) (Fig. 1A). To isolate proteins with net positive surface charges, the conditioned medium was loaded into the cation exchange column (HiTrap SP HP, GE Healthcare BioSciences). The charged proteins were then eluted linearly from the column with 4-morpholineethanesulfonic acid (MES, Sigma-Aldrich) buffer (pH 6) at 0.3 M NaCl (low cation fraction), 0.6 M NaCl (medium cation fraction) and 1 M NaCl (high cation fraction), increased in a linear fashion. To isolate proteins with net negative surface charges, the conditioned medium was loaded into the anion exchange column (HiTrap Q HP, GE Healthcare BioSciences). The charged proteins were then eluted from the column with trisaminomethane (Tris, Sigma-Aldrich) buffer (pH 8) at 0.3 M NaCl (low anion fraction), 0.6 M NaCl (medium anion fraction) and 1 M NaCl (high anion fraction), increased in a linear fashion. The flow rate was set at 1 mL/minute and all experiments were performed at room temperature. The fractionated medium were filtered through a 0.2 µm filter, concentrated by a factor of 50 times (final volume of 200 µL) and desalted using Amicon Ultra-15 centrifugal filter devices with 3000 molecular weight cut off membrane (Millipore, MA, USA).

### Extracellular vesicle isolation

EVs were isolated from conditioned media by ultracentrifugation method. Briefly, conditioned medium was transferred to sterile eppendorf tubes for centrifugation at 10,000 *g* for 20 minutes at 4 °C to remove cells and cell debris. The supernatant were then ultracentrifuged twice at 100,000 *g* for 60 minutes at 4 °C in a MLA-80 rotor (Beckman Coulter, IN, USA) to pellet EVs. The EVs were resuspended in 200 µL of sterile PBS for various bioassays. The EVs and exosomes were characterised using nanoparticle tracking, cryogenic transmission electron microscopy and dissociation-enhanced lanthanide fluorescence immunoassay.

### DELFIA protein quantification

EV proteins were quantified using a previously validated dissociation-enhanced lanthanide fluorescence immunoassay (DELFIA)^43^. Briefly, 50 µL of each sample containing approximately 3.6 µg of proteins was diluted to 100 μL in PBS, added to high-binding ELISA plates, and then incubated overnight at 4 °C. The plates were blocked with 100 μL of PBS containing 1% BSA for 1 hour at room temperature. CD81 antibody (BD Biosciences, Oxford, UK) was added at 1 μg/mL and plates were incubated for 2 hours at room temperature. After washing 3 times, goat anti-rabbit IgG was added (1:2000 in blocking buffer), and incubated for 1 hour at room temperature. Plates were washed 3 times and 1:1000 streptavidin–Europium conjugate in DELFIA Assay Buffer (PerkinElmer, Cambridge, UK) was added and incubated for 1 hour. Finally, 100 µL of the DELFIA Enhancement Solution was added. Time-resolved fluorimetry was performed using a PHERAstar plate reader (BMG Labtech, Ortenberg, Germany) with excitation of 337 nm, detection at 620 nm, integration time set at 200 µs and lag time of 60 µs.

### Cell survival assay

Human cardiac microvascular endothelial cells (HCMECs, Lonza) were plated at 1.5 × 10^4^ cells per well of a 48-well plate and cultured in EGM2-MV medium (Lonza). Neonatal rat cardiomyocytes were plated at 3 × 10^4^ cells/cm^2^ in a 24-well plate pre-coated with fibronectin and cultured in rat cardiomyocyte culture medium. After overnight incubation, cells were washed twice with PBS and cultured in serum-free basal medium (25% endothelial basal medium and 75% M199 medium) supplemented with 20% concentrated serum-free basal medium (as control for unfractionated and fractionated conditioned media), serum-free basal medium supplemented with 5% FCS (as positive control), serum-free basal medium supplemented with 20% concentrated conditioned media (unfractionated or fractionated), serum-free basal medium supplemented with 20% PBS (as control for EVs), or serum-free basal medium supplemented with 20% EVs. To simulate acute ischemic injury, HCMECs were then subjected to hypoxia (<0.1% O_2_ with GENbox AnaerJar, BioMerieux, IL, USA) at 37 °C for 24 hours. Neonatal rat cardiomyocytes were subjected to hypoxia at 37 °C for 6 hours. Cells were then stained with Hoechst 33258 (Sigma-Aldrich) and propidium iodide (Invitrogen). Images at 100x magnification were taken with an inverted microscope (Olympus IX-71 microscope). The number of dead cells (propidium iodide positive) was counted and expressed as a percentage over total number of cells (Hoechst 33258 positive).

### Tube formation assay

Pre-chilled 96-well microplates were coated with 50 µL per well of Growth Factor Reduced Matrigel (BD biosciences) and incubated for 30 min at 37 °C to solidify. HCMECs were washed twice with PBS and re-suspended in serum-free basal medium. 40 µL of cell suspension containing 1 × 10^4^ cells was added to each well containing 40 µL of concentrated serum-free basal medium (as control for unfractionated and fractionated conditioned media), concentrated serum-free basal medium supplemented with 5% FCS (as positive control), concentrated conditioned media (unfractionated or fractionated), PBS (as control for EVs), or EVs. Cells were incubated at 37 °C and the formation of tube-like structures was examined microscopically at 4 hours. Images at 40× magnification were taken with an inverted microscope (Olympus IX-71 microscope) and analysed using ImageJ^4^ and MetaMorph Premier software program (Molecular Devices, CA, USA) in a blinded fashion as previously described^44^. Three images were taken from each well and data were averaged for each parameter.

### Cardiomyocyte proliferation assay

Neonatal rat cardiomyocytes were plated at 1.5 × 10^4^ cells/cm^2^ in a 48-well plate pre-coated with fibronectin and cultured in rat cardiomyocyte culture medium. After overnight incubation, cells were washed twice with PBS and cultured in serum-free basal medium (25% endothelial basal medium and 75% M199 medium) supplemented with 20% concentrated serum-free basal medium (as control for unfractionated and fractionated conditioned media), serum-free basal medium supplemented with 5% FCS (as positive control), serum-free basal medium supplemented with 20% concentrated conditioned media (unfractionated or fractionated), serum-free basal medium supplemented with 20% PBS (as control for EVs), or serum-free basal medium supplemented with 20% EVs. After 24 hours, cells were trypsinised into single cell suspension and spun onto coated glass slide. Cells were fixed and permeabilised. Cell were then incubated with a serum-free blocking solution followed by cardiac troponin T and Ki67 antibodies for an overnight at 4 °C. Cells were then incubated with Alexa Fluor 488 goat anti-rabbit IgG and Alexa Fluor 594 goat anti-mouse IgG for 60 min at room temperature, counterstained with DAPI and mounted with fluorescence mounting agent. Images were taken using a fluorescence microscope (Olympus BX-61 microscope). The number of proliferating cardiomyocytes (cardiac troponin T and Ki67 double positive) was counted and expressed as a percentage over total number of cardiomyocytes (cardiac troponin T positive). Analysis was performed by D.H. in blinded fashion.

### Protein sample preparation and analysis by liquid chromatography coupled with tandem mass spectrometry (LC-MS/MS)

EVs were lysed using radio immune precipitation assay (RIPA) buffer (0.1% SDS, 0.5% Na-DOC, 20 mM HEPES buffer, pH 7.5, 150 mM NaCl, containing protease and phosphatase inhibitor cocktail) and the proteins were precipitated from the supernatant with ice cold acetone at −20 °C overnight. Both proteins from EVs and conditioned media (from single biological sample) were denatured with 8 M urea in 50 mM triethylammonium bicarbonate buffer, reduced in 10 mM tris(2-carboxyethyl)phosphine, alkylated in 55 mM iodoacetamide and digested with trypsin. After clean-up using reverse-phase cartridge (Oasis HLB, Waters, UK), 1 µg of protein digests were analysed by LC-MS/MS using a Q Exactive Plus mass spectrometer (Thermo Fisher Scientific) coupled to an Ultimate 3000 UHPLC (Thermo Fisher Scientific). Solvent A is 0.1% formic acid and solvent B is 0.1% FA in acetonitrile. Each sample was injected onto a PepMap C18 trap column (75 μM X 2 cm, 3 μM, 100 Å, Thermo Fisher Scientific) at 5 μL/minute for 5 minutes using 0.05% trifluoroacetic acid and 3% acetonitrile, and then separated through a PepMap C18 analytical column (75 μM X 50 cm, 2 μM, 100 Å, Thermo Fisher Scientific) at a flow rate of 300 nL/minute. Both columns were maintained at 50 °C. During separation, the percentage of solvent B was increased from 3% to 25% in 35 minutes (for low cation and medium cation fractions) or 180 minutes (for unfractionated conditioned medium and EV fraction), from 25% to 40% in 2 minutes and from 40% to 85% in 2 minutes. The full MS scans were acquired at m/z 375–1400, a resolving power of 70,000, an AGC target value of 3.0 × 10^6^ and a maximum injection time of 50 milliseconds. The top 15 most abundant ions in the MS spectra was subjected to higher-energy collisional dissociation at a resolving power of 35,000, an AGC target value of 1 × 10^5^, a maximum injection time of 120 milliseconds, an isolation window of m/z 1.2 and a normalized collision energy (NCEs) of 30%. Dynamic exclusion of 30 seconds was enabled.

### Proteomics data analysis

Raw data were searched using MaxQuant software (1.5.3.30) against SwissProt human database (20,193 entries). Enzyme specificity was set to trypsin, allowing for cleavage at N-terminal to proline and up to 2 missed cleavage. Carbamidomethyl cysteine as fixed modification and oxidized methionine and protein N-acetylation as variable modifications were set. The false discovery rate (FDR) for peptide and protein identification was set to 1%. After removing common laboratory contaminant proteins, at least 2 unique/razor peptides were required for protein identification. Functional enrichment analysis was performed with FunRich using FunRich human database, and statistically analysed with hypergeometric test using FunRich human genome database as the background^16^. A p value < 0.05 indicates a sub-group of genes that is significantly enriched in the sample against the background genome. The protein interaction map was also generated by FunRich.

### RNA isolation and purification

Total RNA from EVs was extracted using mirVana miRNA isolation kit (Thermo Fisher Scientific). Total RNA samples was then purified with magnetic beads (Beckman Coulter) followed by library preparation using an input weight of 445 ng of total RNA using TruSeq Stranded mRNA Library Kit (Illumina, CA, USA) with omission of the mRNA isolation step.

### RNA sequencing

The indexed libraries were pooled and prepared for paired end sequencing on a NextSeq. 500 instrument using the 150 cycle kit v2 chemistry (Illumina) as per manufacturer’s instructions. The parameters were 2 × 75 paired end bases of sequencing with a 6 base index read to separate the reads into the specific libraries. The base calling and quality scoring were determined using Real-Time Analysis on board software v2.4.6, while the FASTQ file generation and de-multiplexing utilised bcl2fastq conversion software v2.15.0.4.

### RNA sequencing bioinformatics analysis

The RNA sequencing data consist of 47,121,770 paired-end reads where each read is 80 bp long. FastQC (https://www.bioinformatics.babraham.ac.uk/projects/fastqc/) was used to check the quality of the reads. On average the read quality was uniformly high throughout the length of the 80 bp reads, except for the first 5 bp and last 5 bp where the base quality was lower. Thus, the first and last 5 bp of each read were trimmed away to ensure good sequence alignment can be achieved. FastQC analysis of the trimmed data confirmed that the trimming was successful in terms of removing low quality base pairs. STAR^45^ was then employed to align the trimmed reads against the human reference genome hg38, using STAR’s default parameters. Of the 47 million reads, almost all the reads (99%) can be mapped to the genome. Nonetheless, only ~2% of the reads (1.27 million) can be uniquely aligned to the genome (i.e., one read is only mapped to one locus in the genome), while the remaining ~97% of the reads can be mapped to multiple loci in the genome. To confirm our analysis results, a newer RNA-seq aligner, HISAT2^46^, was employed to align the trimmed RNA sequencing reads. Similarly, HISAT reported ~2% of the reads were uniquely mapped and most of the remaining reads were mapped to more than one genomic loci.

A read summarization program, featureCounts^47^, was employed to quantify the number of RNA sequencing paired-end reads that were mapped to individual genomic features. In particular, feature quantification was performed using both uniquely mappable reads only (Unique) and all reads that are mappable to the genome where each read is represented by one ‘best’ alignment (Primary). Read count was quantified against GENCODE gene annotation (Release 26)^48^. Each transcript can be classified as protein-coding genes, short non-coding RNA, long non-coding RNA, pseudogene and other transcripts.

The expression of a transcript was represented as sequence Fragment Per Million (FPM). A read count of 1 was considered as a transcript to be expressed. Gene expression measured by log2(FPM+1) identified by Unique and Primary was highly correlated (Supplementary Fig. S7), suggesting that the overall gene expression profile was similar despite a big difference in terms of total read count (1.27 million Unique versus 47 million in Primary). Furthermore, gene expression profiles measured by STAR- and HISAT-aligned reads were strongly correlated regardless whether Unique or Primary alignments were used. STAR uniquely-mapped reads were likely give the most conservative gene expression estimates and were therefore selected for all downstream analyses. g:Profiler^49^ was employed for Gene Ontology (GO) and pathway term analyses with the following parameters: minimal set size of 5, maximal set size of 500, minimal intersect size of 2, p value was corrected by FDR and the IEA results were excluded. Both KEGG term and Reactome term were also included in the pathway analysis. For the GO and pathway heat map, -log10(FDR) were calculated for the matrix and top 5 terms from each category were selected.

To identify a set of high confidence and expressed miRNA set in the EVs, miRNAs with at least two mapped reads around the miRNA precursor regions in the primary alignment were selected from the RNA sequencing data. Among these expressed miRNA, the RNA sequencing data was manually inspected at every miRNA location in a genome browser to ensure that the reads were mapped to one unique miRNA, were of high mapping quality and were not likely a results of alignment to nearby transcripts. The target genes of each miRNA were predicted using TargetScan (v7.1)^17^. Predicted target genes that have a ‘cumulative weighted context++ score’^17^ of less than −0.3 were selected to ensure that only high confident predicted target genes were included. g:Profiler^49^ was then employed for GO terms and pathway analyses.

### Statistical analysis

Data are expressed as mean ± SEM. Statistical analysis was performed on Graphpad Prism software. The significance of the differences was evaluated using the Student’s *t* test or one-way analysis of variance (ANOVA) followed by Bonferroni multiple-comparison *post hoc* analysis where appropriated. Values of p < 0.05 were considered statistically significant.

All data generated or analysed during this study are included in this published article (and its Supplementary Information files).

## Electronic supplementary material

Supplementary Information

Supplementary Dataset 1

Supplementary Dataset 2

Supplementary Dataset 3

Supplementary Dataset 4
