# Supplementary material for: Biologically active constituents of the secretome of human W8B2+ cardiac stem cells
Source: Sci Rep. 2018 Jan 25;8:1579. doi: 10.1038/s41598-018-19855-4 (PMC5785502; doi:10.1038/s41598-018-19855-4)
Supplement: Supplementary file 1 — Supplementary Information [file 41598_2018_19855_MOESM1_ESM.pdf]

Supplementary Information

Biologically active constituents of the secretome of human W8B2$^+$ cardiac stem cells

Shuai Nie, Xin Wang, Priyadharshini Sivakumaran, Mark MW Chong, Xin Liu, Tara Karnezis, Nadeeka Bandara, Kaloyan Takov, Cameron J Nowell, Stephen Wilcox, Mitch Shambrook, Andrew F Hill, Nicole C Harris, Andrew E Newcomb, Padraig Strappe, Ramin Shayan, Damián Hernández, Jordan Clarke, Eric Hanssen, Sean Davidson, Gregory J Dusting, Alice Pébay, Joshua WK Ho, Nicholas Williamson, Shiang Y Lim

Methods

All experiments were performed in accordance with relevant guidelines and regulations of Australian National Health and Medical Research Council.

W8B2$^+$ cardiac stem cells

W8B2$^+$ CSCs were isolated from atrial appendages as previously described[1]. Human tissues were collected with informed consent according to the Australian National Health and Medical Research Council guidelines and with approval from the Human Research Ethics Committee-A of St. Vincent's Hospital (HREC-A 07/08). Briefly, atrial appendages obtained from consented adult patient underwent coronary artery bypass grafting were minced into small fragments and subjected to brief collagenase digestion. The tissue fragments were then seeded onto fibronectin-coated plated and cultured in explant medium containing IMDM supplemented with 20% fetal calf serum (Sigma-Aldrich, MO, USA), 0.1 mM β-mercaptoethanol (Thermo Fisher Scientific, MA, USA), 2 mM L-glutamine (Thermo Fisher Scientific), 100 U/mL penicillin (Lonza, MD, USA), 100 µg/mL streptomycin (Lonza) and 0.25 µg/mL amphotericin B (Lonza). The resulting monolayer of outgrowth cells were then collected and expanded in growth medium containing endothelial growth medium (EGM-2) and M199 medium (Lonza), in 1:3 ratio, supplemented with 10% fetal calf serum, 0.1 mM non-essential amino acids (Thermo Fisher Scientific), 100 U/mL penicillin, 100 µg/mL streptomycin and 0.25 µg/mL amphotericin B. Once confluent, cells were enriched for W8B2$^+$ cells by staining with the W8B2 PE-conjugated antibody (mouse anti-human IgG1; Biolegends, CA, USA), sorted using a Flow cytometer and sorter (FACSAria, BD Biosciences, USA) and expanded in growth medium as passage 1.

Isolation of neonatal rat cardiomyocytes

Neonatal rat cardiomyocytes were isolated from the ventricles of 1-3 days old Sprague-Dawley rats

according to the manufacture's procedure (Neonatal Cardiomyocyte Isolation System, Worthington Biochemical, NJ, USA). Isolated cardiomyocytes were suspended with rat cardiomyocyte culture medium containing DMEM/F12 medium (Thermo Fisher Scientific), 5% horse serum (Thermo Fisher Scientific), 1% antibiotic/antimycotic solution (Thermo Fisher Scientific), 100 μg/mL ampicillin (Thermo Fisher Scientific), 3 mM pyruvic acid (Sigma-Aldrich), 2 mg/mL bovine serum albumin (Sigma-Aldrich), insulin-transferrin-selenium solution (20 ng/mL, 11 ng/mL, 13.4 pg/mL respectively) (Thermo Fisher Scientific), 5 μg/mL linoleic acid (Sigma-Aldrich) and 100 μM ascorbic acid (Sigma-Aldrich). Cells were pre-plated twice on uncoated petri dishes for 20 minutes each at 37^0C to remove non-myocytes. Enriched cardiomyocytes were then seeded onto cell culture plates pre-coated with 10 μg/mL of fibronectin and cultured at 37^0C in a humidified atmosphere containing 5% CO_2.

Collection of conditioned media

$W8B2^+$ CSCs (passage 2-4) were cultured to 95% confluence in T75 culture flask, washed twice with PBS, and replenished with 10 mL of serum-free basal medium (25% endothelial basal medium and 75% M199 medium) for 72 hours at 37°C in a humidified incubator containing 5% CO_2. Collected media were filtered through a 0.2 μm filter, concentrated by a factor of 50 times (final volume of 200 μL) and desalted using Amicon Ultra-15 centrifugal filter devices with 3000 molecular weight cut off membrane (Millipore, MA, USA).

Ion exchange chromatography

Charged proteins in the conditioned medium harvested from $W8B2^+$ CSCs were separated and purified by cation and anion exchange chromatography (HiTrap SP and Q, 1 mL column, GE Healthcare BioSciences, Sweden) using BioLogic LP low-pressure chromatography system (Bio-Rad, CA, USA) (Figure 1A). To isolate proteins with net positive surface charges, the conditioned medium was loaded into the cation exchange column (HiTrap SP HP, GE Healthcare BioSciences). The charged proteins were then eluted linearly from the column with 4-morpholineethanesulfonic acid (MES, Sigma-Aldrich) buffer (pH 6) at 0.3 M NaCl (low cation fraction), 0.6 M NaCl (medium cation fraction) and 1 M NaCl (high cation fraction), increased in a linear fashion. To isolate proteins with net negative surface charges, the conditioned medium was loaded into the anion exchange column (HiTrap Q HP, GE Healthcare BioSciences). The charged proteins were then eluted from the column with trisaminomethane (Tris, Sigma-Aldrich) buffer (pH 8) at 0.3 M NaCl (low anion fraction), 0.6 M NaCl (medium anion fraction) and 1 M NaCl (high anion fraction), increased in a linear fashion. The flow rate was set at 1 mL/minute

and all experiments were performed at room temperature. The fractionated medium were filtered through a 0.2 µm filter, concentrated by a factor of 50 times (final volume of 200 µL) and desalted using Amicon Ultra-15 centrifugal filter devices with 3000 molecular weight cut off membrane (Millipore, MA, USA).

Extracellular vesicle isolation

EVs were isolated from conditioned media by ultracentrifugation method. Briefly, conditioned medium was transferred to sterile eppendorf tubes for centrifugation at 10,000*g* for 20 minutes at 4°C to remove cells and cell debris. The supernatant were then ultracentrifuged twice at 100,000*g* for 60 minutes at 4°C in a MLA-80 rotor (Beckman Coulter, IN, USA) to pellet EVs. The EVs were resuspended in 200 µL of sterile PBS for various bioassays. The EVs and exosomes were characterised using nanoparticle tracking, electron microscopy and dissociation-enhanced lanthanide fluorescence immunoassay (DELFIA).

Nanoparticle tracking analysis

The size of EVs was examined using the NS300 Nanosight (Malvern Instruments Ltd, Malvern, UK) with constant flow injection. EVs (suspended in PBS and stored at 4°C overnight) were analysed at camera level of 5, detection threshold of 4, slider shutter of 45 and slider grain of 15. Five recordings of 30 seconds each were captured and the data from at least 5,000 individual particle tracks were analysed per sample.

Cryogenic transmission electron microscopy

Freshly isolated EVs and exosomes in distilled water were deposited onto glow-discharged C-flat holey carbon grids (ProSciTech, Queensland, Australia). After blotting off excess liquid, the grid were plunged-frozen in liquid ethane using a Vitrobot (FEI, OR, USA). Images were acquired at 300 kV using a TecnaiG2 F30 transmission electron microscopy (FEI).

DELFIA protein quantification

EV proteins were quantified using a previously validated dissociation-enhanced lanthanide fluorescence immunoassay (DELFIA)[2]. Briefly, lyophilised sample was resuspended in 400 µL of PBS and protein concentration was determined using Low-concentration BCA protein assay kit (Abcam, Cambridge, UK). 50 µL of each sample containing approximately 3.6 µg of proteins was diluted to 100 µL in PBS, added to high-binding ELISA plates, and then incubated overnight at 4°C. The plates were washed 3 times with DELFIA wash buffer (PerkinElmer, Cambridge, UK). Wells were blocked with 100 µL of

PBS containing 1% BSA for 1 hour at room temperature, then washed 3 times. CD81 antibody (clone JS-81; BD Biosciences, Oxford, UK) was added at 1 µg/mL and plates were incubated for 2 hours at room temperature. After washing 3 times, goat anti-rabbit IgG was added (1:2000 in blocking buffer), and incubated for 1 hour at room temperature. Plates were washed 3 times and 1:1000 streptavidin–Europium conjugate in DELFIA Assay Buffer (PerkinElmer) was added and incubated for 1 hour. Finally, after 6 washes, 100 µL of the DELFIA Enhancement Solution was added and the plate was shaken for 5 minutes twice on the plate reader. Time-resolved fluorimetry was performed using a PHERAstar plate reader (BMG Labtech, Ortenberg, Germany) with excitation of 337 nm, detection at 620 nm, integration time set at 200 µs and lag time of 60 µs.

Cell survival assay

Human cardiac microvascular endothelial cells (HCMECs, Lonza) were plated at 1.5×10^4 cells per well of a 48-well plate and cultured in EGM2-MV medium (Lonza). Neonatal rat cardiomyocytes were plated at 3×10^4 cells/cm^2 in a 24-well plate pre-coated with fibronectin and cultured in rat cardiomyocyte culture medium. After overnight incubation, cells were washed twice with PBS and cultured in serum-free basal medium (25% endothelial basal medium and 75% M199 medium) supplemented with 20% concentrated serum-free basal medium (as control for unfractionated and fractionated conditioned media), serum-free basal medium supplemented with 5% FCS (as positive control), serum-free basal medium supplemented with 20% concentrated conditioned media (unfractionated or fractionated), serum-free basal medium supplemented with 20% PBS (as control for EVs), or serum-free basal medium supplemented with 20% EVs. To simulate acute ischemic injury, HCMECs were then subjected to hypoxia (<0.1% O_2 with GENbox AnaerJar, BioMerieux, IL, USA) at 37^0C for 24 hours. Neonatal rat cardiomyocytes were subjected to hypoxia at 37^0C for 6 hours. Cells were then stained with Hoechst 33258 (Sigma-Aldrich) and propidium iodide (Invitrogen). Images at 100x magnification were taken with an inverted microscope (Olympus IX-71 microscope). The number of dead cells (propidium iodide positive) was counted and expressed as a percentage over total number of cells (Hoechst 33258 positive).

Tube formation assay

Pre-chilled 96-well microplates were coated with 50 µL per well of Growth Factor Reduced Matrigel (BD biosciences) and incubated for 30 min at 37^0C to solidify. HCMECs were washed twice with PBS and re-suspended in serum-free basal medium. 40 µL of cell suspension containing 1×10^4 cells was added to each well containing 40 µL of concentrated serum-free basal medium (as control for unfractionated

and fractionated conditioned media), concentrated serum-free basal medium supplemented with 5% FCS (as positive control), concentrated conditioned media (unfractionated or fractionated), PBS (as control for EVs), or EVs. Cells were incubated at 37^{0}C and the formation of tube-like structures was examined microscopically at 4 hours. Images at 40x magnification were taken with an inverted microscope (Olympus IX-71 microscope) and analysed using ImageJ[4] and MetaMorph Premier software program (Molecular Devices, CA, USA) in a blinded fashion as previously described[43]. Three images were taken from each well and data were averaged for each parameter.

Cardiomyocyte proliferation assay

Neonatal rat cardiomyocytes were plated at 1.5x10^4 cells/cm^2 in a 48-well plate pre-coated with fibronectin and cultured in rat cardiomyocyte culture medium. After overnight incubation, cells were washed twice with PBS and cultured in serum-free basal medium (25% endothelial basal medium and 75% M199 medium) supplemented with 20% concentrated serum-free basal medium (as control for unfractionated and fractionated conditioned media), serum-free basal medium supplemented with 5% FCS (as positive control), serum-free basal medium supplemented with 20% concentrated conditioned media (unfractionated or fractionated), serum-free basal medium supplemented with 20% PBS (as control for EVs), or serum-free basal medium supplemented with 20% EVs. After 24 hours, cells were trypsinised into single cell suspension and spun onto coated glass slide. Cells were fixed and permeabilised. Cell were then incubated with a serum-free blocking solution followed by cardiac troponin T and Ki67 antibodies for an overnight at 4°C. Cells were then incubated with Alexa Fluor 488 goat anti-rabbit IgG and Alexa Fluor 594 goat anti-mouse IgG for 60 min at room temperature, counterstained with DAPI and mounted with fluorescence mounting agent. Images were taken using a fluorescence microscope (Olympus BX-61 microscope). The number of proliferating cardiomyocytes (cardiac troponin T and Ki67 double positive) was counted and expressed as a percentage over total number of cardiomyocytes (cardiac troponin T positive). Analysis was performed by D.H. in blinded fashion.

Protein sample preparation and analysis by liquid chromatography coupled with tandem mass spectrometry (LC-MS/MS)

EVs were lysed using radio immune precipitation assay (RIPA) buffer (0.1% SDS, 0.5% Na-DOC, 20 mM HEPES buffer, pH 7.5, 150 mM NaCl, containing protease and phosphatase inhibitor cocktail) and the proteins were precipitated from the supernatant with ice cold acetone at -20°C overnight. After

protein concentration measurement using BCA assay (Thermo Fisher Scientific), both proteins from EVs and conditioned media (from single biological sample) were denatured with 8 M urea in 50 mM triethylammonium bicarbonate buffer, reduced in 10 mM tris(2-carboxyethyl)phosphine, alkylated in 55 mM iodoacetamide and digested with trypsin. After clean-up using reverse-phase cartridge (Oasis HLB, Waters, UK), 1 µg of protein digests were analysed by LC-MS/MS using a Q Exactive Plus mass spectrometer (Thermo Fisher Scientific) coupled to an Ultimate 3000 UHPLC (Thermo Fisher Scientific). Solvent A is 0.1% formic acid and solvent B is 0.1% formic acid in acetonitrile. Each sample was injected onto a PepMap C18 trap column (75 µM X 2 cm, 3 µM, 100 Å, Thermo Fisher Scientific) at 5 µL/minute for 5 minutes using 0.05% trifluoroacetic acid and 3% acetonitrile, and then separated through a PepMap C18 analytical column (75 µM X 50 cm, 2 µM, 100 Å, Thermo Fisher Scientific) at a flow rate of 300 nL/minute. Both columns were maintained at 50°C. During separation, the percentage of solvent B was increased from 3% to 25% in 35 minutes (for low cation and medium cation fractions) or 180 minutes (for unfractionated conditioned medium and EV fraction), from 25% to 40% in 2 minutes and from 40% to 85% in 2 minutes. The full MS scans were acquired at m/z 375 – 1400, a resolving power of 70,000, an AGC target value of 3.0×10^6 and a maximum injection time of 50 milliseconds. The top 15 most abundant ions in the MS spectra was subjected to higher-energy collisional dissociation at a resolving power of 35,000, an AGC target value of 1×10^5, a maximum injection time of 120 milliseconds, an isolation window of m/z 1.2 and a normalized collision energy (NCEs) of 30%. Dynamic exclusion of 30 seconds was enabled.

Proteomics data analysis

Raw data were searched using MaxQuant software (1.5.3.30) against SwissProt human database (20,193 entries). Enzyme specificity was set to trypsin, allowing for cleavage at N-terminal to proline and up to 2 missed cleavage. Carbamidomethyl cysteine as fixed modification and oxidized methionine and protein N-acetylation as variable modifications were set. The false discovery rate (FDR) for peptide and protein identification was set to 1%. After removing common laboratory contaminant proteins, at least 2 unique/razor peptides were required for protein identification. Functional enrichment analysis was performed with FunRich using FunRich human database, and statistically analysed with hypergeometric test using FunRich human genome database as the background[4]. A p value <0.05 indicates a sub-group of genes that is significantly enriched in the sample against the background genome. The protein interaction map was also generated by FunRich.

RNA isolation and purification

Total RNA from EVs was extracted using mirVana miRNA isolation kit (Thermo Fisher Scientific), and the RNA integrity and quantity were assessed using a RNA tape on the Tapestation (Agilent, CA, USA). Total RNA samples was then purified with magnetic beads (Beckman Coulter) followed by library preparation using an input weight of 445 ng of total RNA using TruSeq Stranded mRNA Library Kit (Illumina, CA, USA) with omission of the mRNA isolation step.

cDNA synthesis and library preparation

RNA fragmentation was performed directly on total RNA by adding Prime Fragment Mix (Illumina) and the fragmented RNA was subjected to cDNA synthesis using TruSeq Stranded mRNA Kit (Illumina) according to manufacturer's protocol. Briefly, the first strand cDNA was synthesized from fragmented RNA using reverse transcriptase (Super-Script II, Invitrogen) and random hexamer primers. The second-strand cDNA was then synthesized using a second strand cDNA synthesis master mix (Illumina). Short DNA fragments were then removed using NGS beads (Macherey-Nagel, Düren, Germany) and the resulting dsDNA was used for library preparation.

The purified fragments were subjected to dA-tailing and ligated with sequencing adapters using TruSeq library preparation kit (Illumina) according to the manufacturer's protocol. Finally, the fragments were enriched by polymerase chain reaction amplification using primer cocktail mix (Illumina) and High fidelity DNA polymerase (Kapa Biosystems, MA, USA) according to the manufacturer's protocol. PCR products were purified with magnetic beads and dissolved in 30 μL of nuclease free water. The library integrity and quantity of enriched libraries were determined using a D1000 tape on the Tapestation.

RNA sequencing

The indexed libraries were pooled and prepared for paired end sequencing on a NextSeq500 instrument using the 150 cycle kit v2 chemistry (Illumina) as per manufacturer's instructions. The parameters were 2 x 75 paired end bases of sequencing with a 6 base index read to separate the reads into the specific libraries. The base calling and quality scoring were determined using Real-Time Analysis on board software v2.4.6, while the FASTQ file generation and de-multiplexing utilised bcl2fastq conversion software v2.15.0.4.

RNA sequencing bioinformatics analysis

The RNA sequencing data consist of 47,121,770 paired-end reads, where each read is 80 bp long. FastQC (https://www.bioinformatics.babraham.ac.uk/projects/fastqc/) was used to check the quality of the reads. On average the read quality was uniformly high throughout the length of the 80 bp reads, except for the first 5 bp and last 5 bp where the base quality was lower. Thus, the first and last 5 bp of each read were trimmed away to ensure good sequence alignment can be achieved. FastQC analysis of the trimmed data confirmed that the trimming was successful in terms of removing low quality base pairs. STAR[5] was then employed to align the trimmed reads against the human reference genome hg38, using STAR's default parameters. Of the 47 million reads, almost all the reads (99%) can be mapped to the genome. Nonetheless, only ~2% of the reads (1.27 million) can be uniquely aligned to the genome (i.e., one read is only mapped to one locus in the genome), while the remaining ~97% of the reads can be mapped to multiple loci in the genome. To confirm our analysis results, a newer RNA-seq aligner, HISAT2[6], was employed to align the trimmed RNA sequencing reads. Similarly, HISAT reported ~2% of the reads were uniquely mapped and most of the remaining reads were mapped to more than one genomic loci.

A read summarization program, featureCounts[7], was employed to quantify the number of RNA sequencing paired-end reads that were mapped to individual genomic features. In particular, feature quantification was performed using both uniquely mappable reads only (Unique) and all reads that are mappable to the genome where each read is represented by one 'best' alignment (Primary). Read count was quantified against GENCODE gene annotation (Release 26)[8]. Each transcript can be classified as protein-coding genes, short non-coding RNA, long non-coding RNA, pseudogene and other transcripts.

The expression of a transcript was represented as sequence Fragment Per Million (FPM). A read count of 1 was considered as a transcript to be expressed. Gene expression measured by log2(FPM+1) identified by Unique and Primary was highly correlated (Supplementary Figure 7), suggesting that the overall gene expression profile was similar despite a big difference in terms of total read count (1.27 million Unique versus 47 million in Primary). Furthermore, gene expression profiles measured by STAR- and HISAT-aligned reads were strongly correlated regardless whether Unique or Primary alignments were used. STAR uniquely-mapped reads were likely give the most conservative gene expression estimates and were therefore selected for all downstream analyses. g:Profiler [9] was employed for Gene Ontology (GO) and pathway term analyses with the following parameters: minimal set size of 5, maximal set size of 500, minimal intersect size of 2, p value was corrected by FDR and the IEA results were excluded. Both KEGG term and Reactome term were also included in the pathway analysis. For the GO

and pathway heat map, -log10(FDR) were calculated for the matrix and Top 5 terms from each category were selected.

microRNA detection and target gene analysis

To identify a set of high confidence and expressed miRNA set in the EVs, miRNAs with at least two mapped reads around the miRNA precursor regions in the primary alignment were selected from the RNA sequencing data. Among these expressed miRNA, the RNA sequencing data was manually inspected at every miRNA location in a genome browser to ensure that the reads were mapped to one unique miRNA, were of high mapping quality and were not likely a results of alignment to nearby transcripts. The target genes of each miRNA were predicted using TargetScan (v7.1)[10]. Predicted target genes that have a 'cumulative weighted context++ score'[10] of less than -0.3 were selected to ensure that only high confident predicted target genes were included. g:Profiler[9] was then employed for GO terms and pathway analyses with the following parameters: minimal set size of 5, maximal set size of 500, minimal intersect size of 2, p value was corrected by FDR and the IEA results were excluded.

Reverse transcription polymerase chain reaction (RT-PCR)

Total RNA from EVs and W8B2$^+$ CSCs was extracted using mirVana miRNA isolation kit (Thermo Fisher Scientific) and the cDNA was synthesized with 58 ng of RNA using a high-capacity cDNA reverse transcription kit (Applied Biosystems, CA, USA). Following cDNA synthesis, polymerase chain reaction was performed to detect mRNAs and microRNA precursors using GoTaq Green Master Mix (Promega, WI, USA) and then subjected to 2% agarose gel electrophoresis. Primer sequences are as follow:

Targets	Sequences (5′–3′)	Size (bp)/ annealing temperature °C/ no. of cycles
MT-RNR1	F: AGACCCAAACTGGGATTAGATAC<br>R: TTAAGCTGTGGCTCGTAGTG	100 / 55 / 35
MT-RNR2	F: GCTCAACACCCACTACCTAAA<br>R: GCTTATGCGGAGGAGAATGT	132 / 55 / 35
MIR1244	F:ATCTTATTCCGAGCATTCCAGT<br>R:TTAACCATCTCATACAAACCA	82 / 56 / 35
MIR3648/3687	F:CTGAGAAACGGCTACCACATC<br>R:GCCTCGAAAGAGTCCTGTATTG	107 / 56 / 35

Supplemental Figures

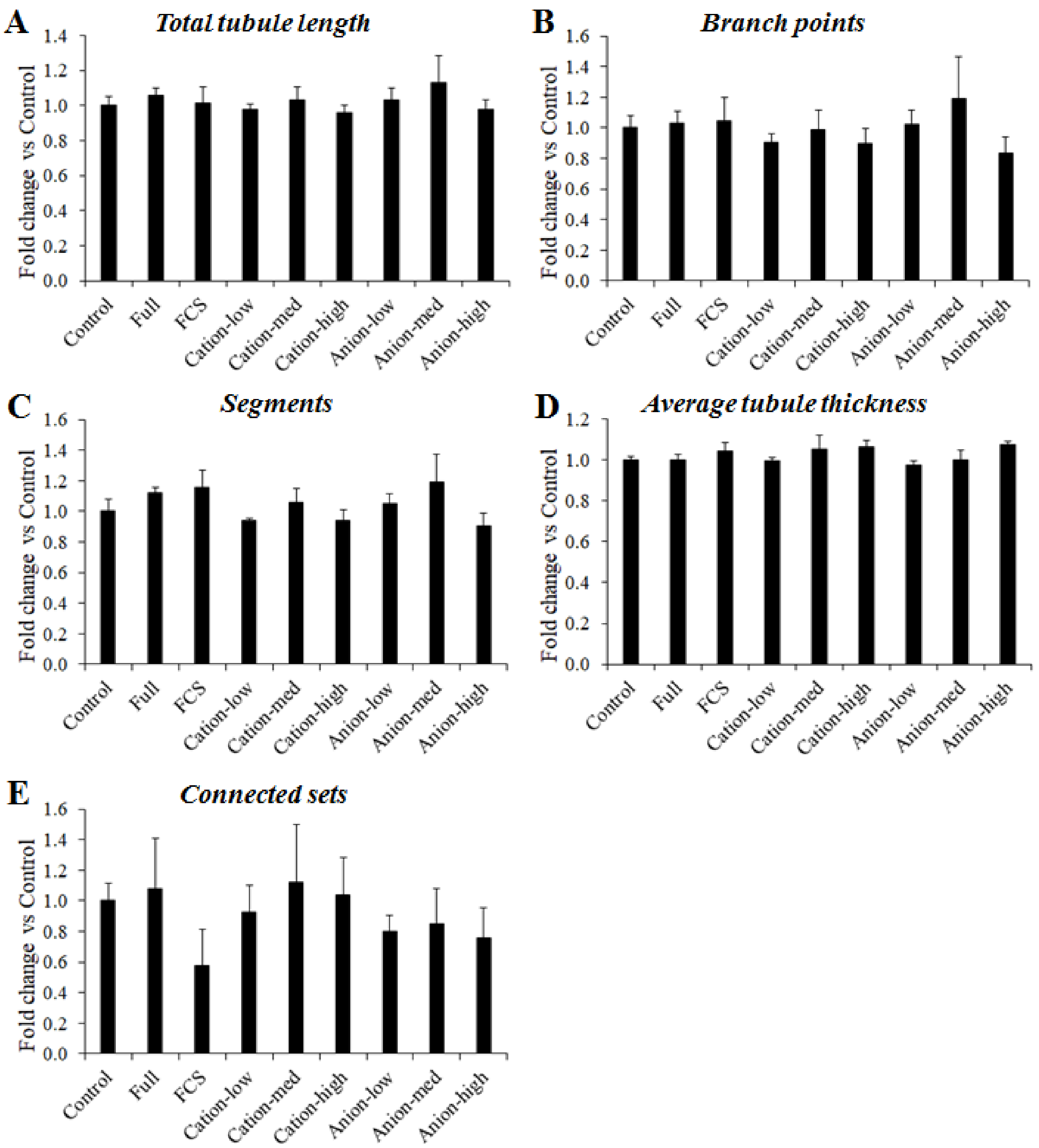

Supplementary Figure S1. The effect of conditioned medium on tube formation of human microvascular endothelial cell. Tube formation was assessed by the total tubule length (**A**), branch points (**B**), segments (**C**), average tubule thickness (**D**) and connected sets (**E**). Data are shown as mean±SEM from 4 independent experiments.

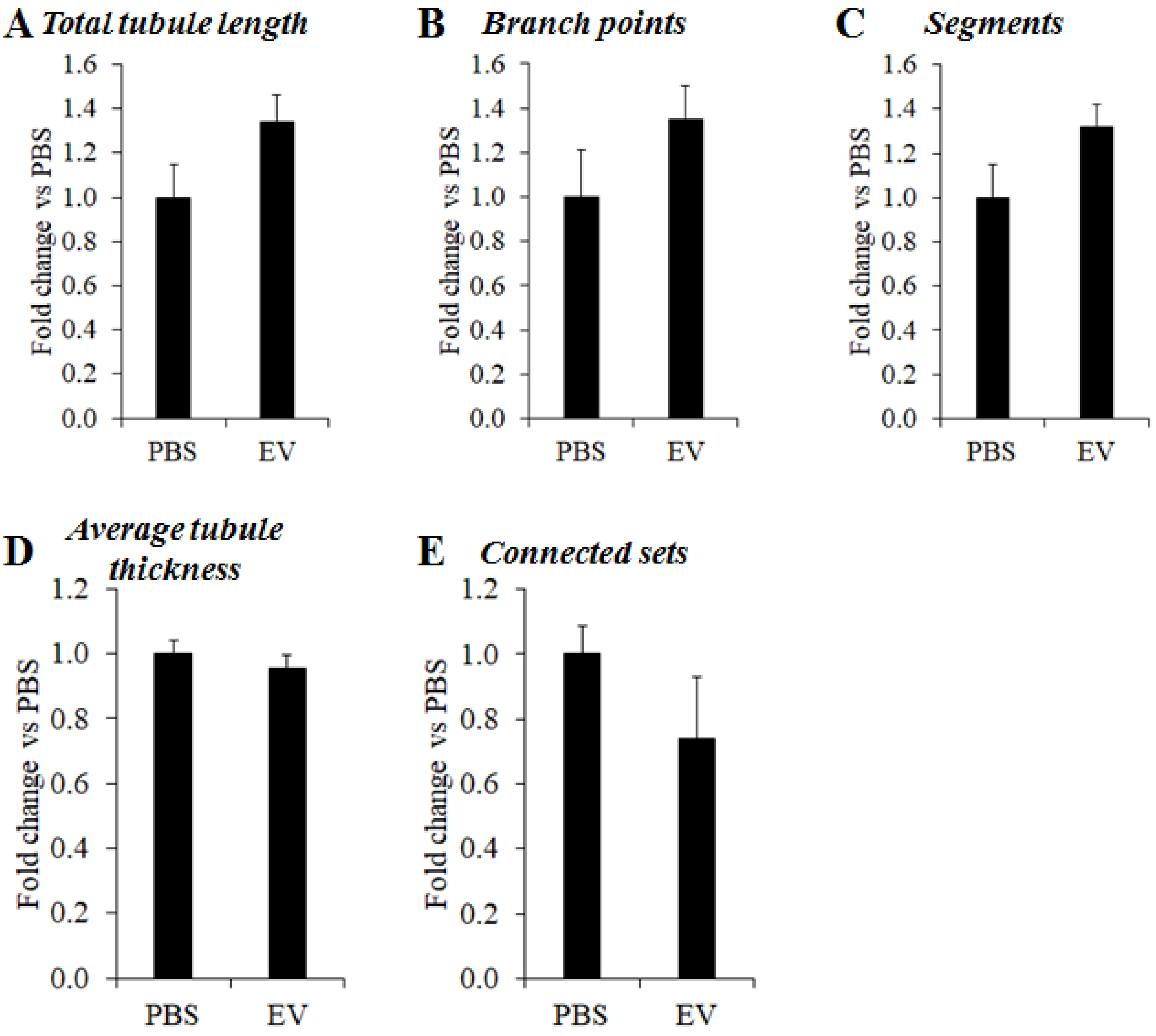

Supplementary Figure S2. The effect of extracellular vesicle fractions of W8B2^{+} CSC conditioned medium on tube formation of human microvascular endothelial cell. Tube formation was assessed by the total tubule length (**A**), branch points (**B**), segments (**C**), average tubule thickness (**D**) and connected sets (**E**). Data are shown as mean±SEM from 4 independent experiments. PBS, phosphate-buffered saline; EV, extracellular vesicles.

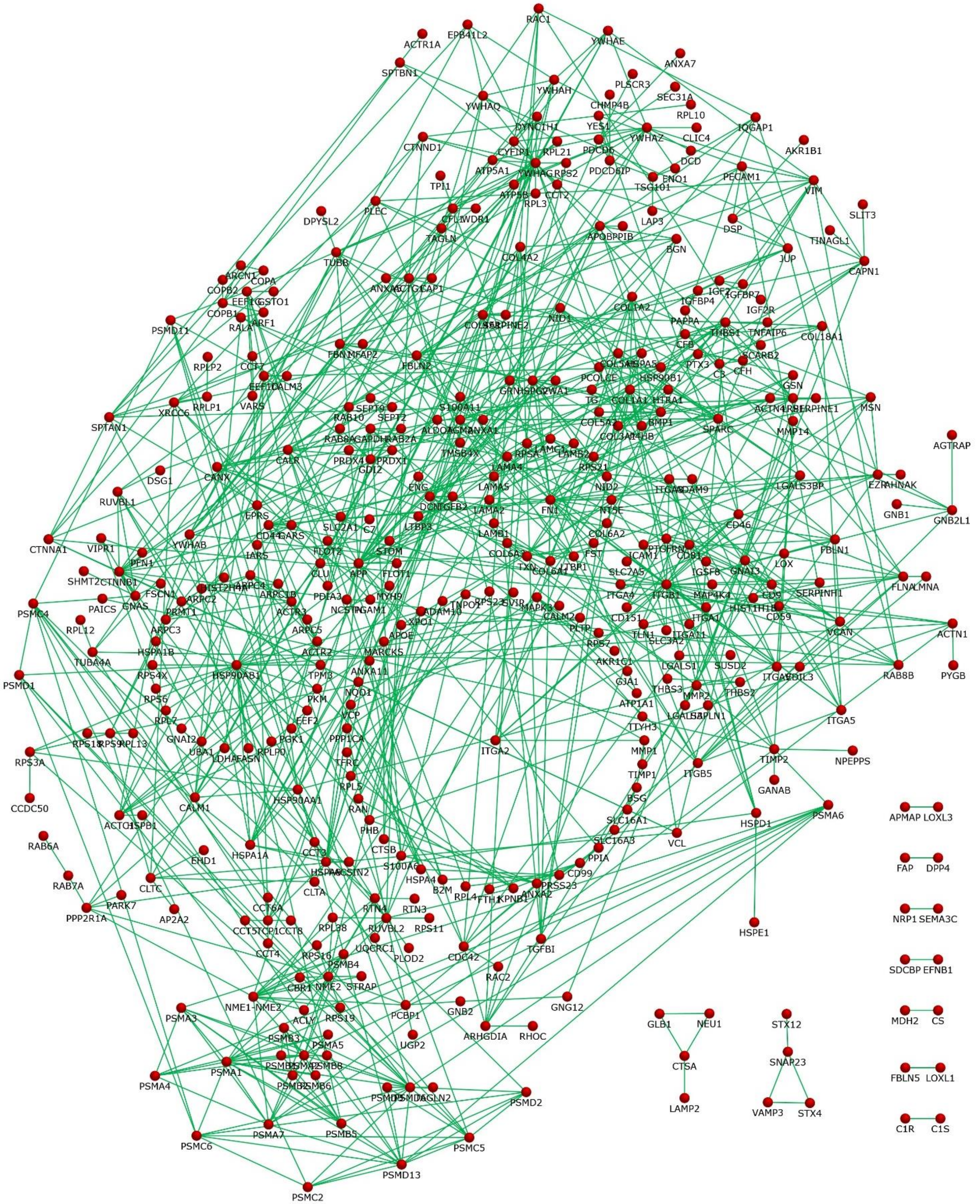

Supplementary Figure S3. An interaction network of proteins identified in extracellular vesicles derived from W8B2^{+} CSCs.

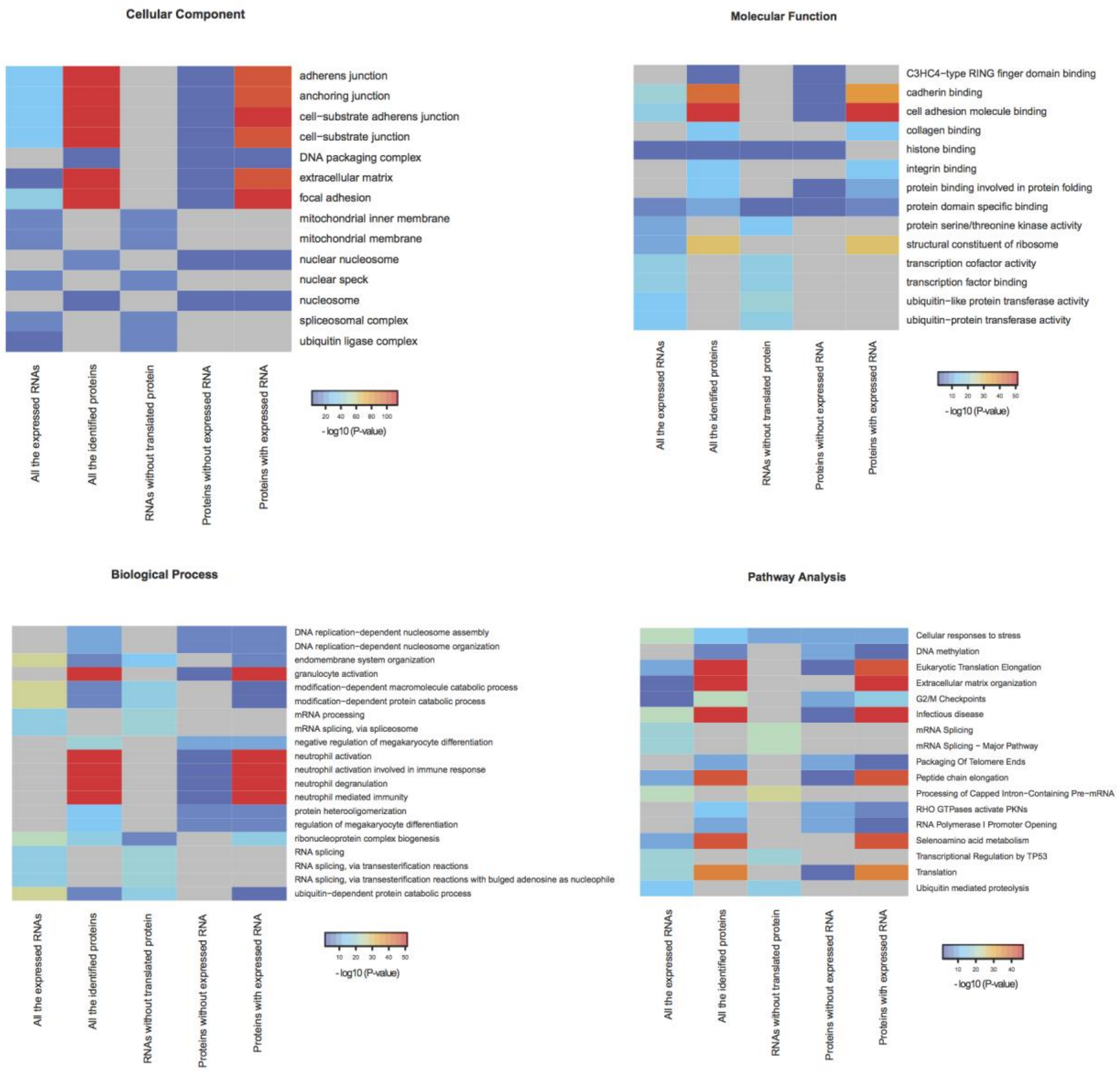

Supplementary Figure S4. Gene Ontology (GO) and pathway analysis on extracellular vesicle proteins and RNA. The cellular component (**A**), molecular function (**B**), biological process (**C**) and pathway analysis (**D**) of all the expressed mRNAs, all the identified proteins, expressed mRNAs that do not overlap with proteins, proteins that do not overlap with expressed mRNA, and proteins that overlap with expressed mRNA are represented by heat maps showing the top 5 GO terms or pathways that are statistically significantly enriched. Grey colour of the heat maps indicates FDR > 0.05.

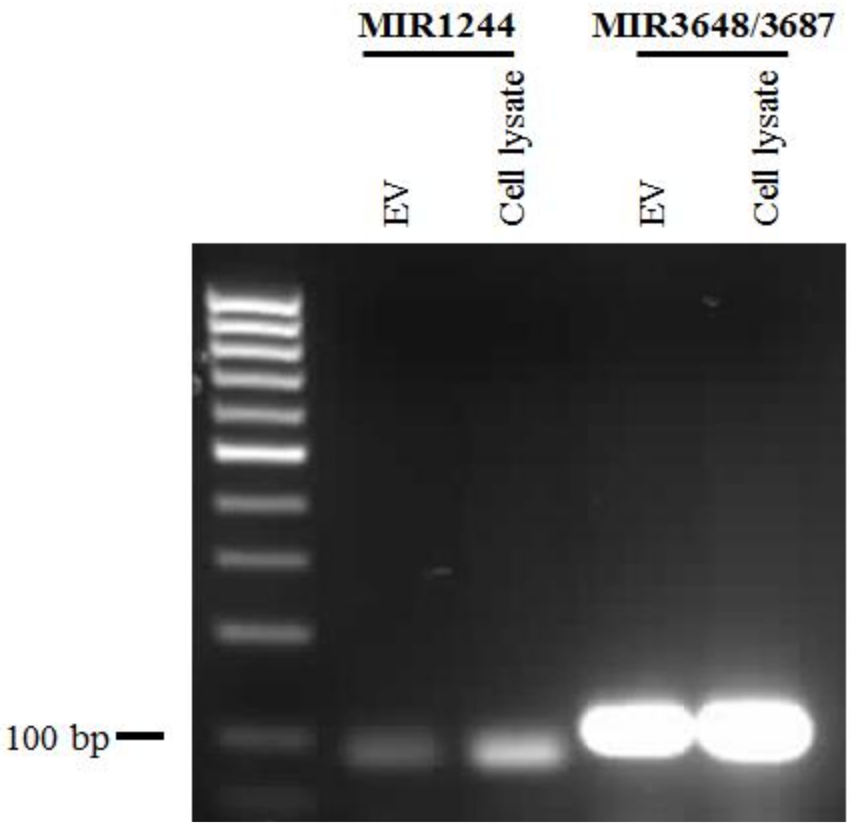

Supplementary Figure S5. PCR analysis of miRNA-1244 and miRNA-3648/3687 precursors in the extracellular vesicles (EV) and whole cell lysate of W8B2$^+$ CSCs.

A

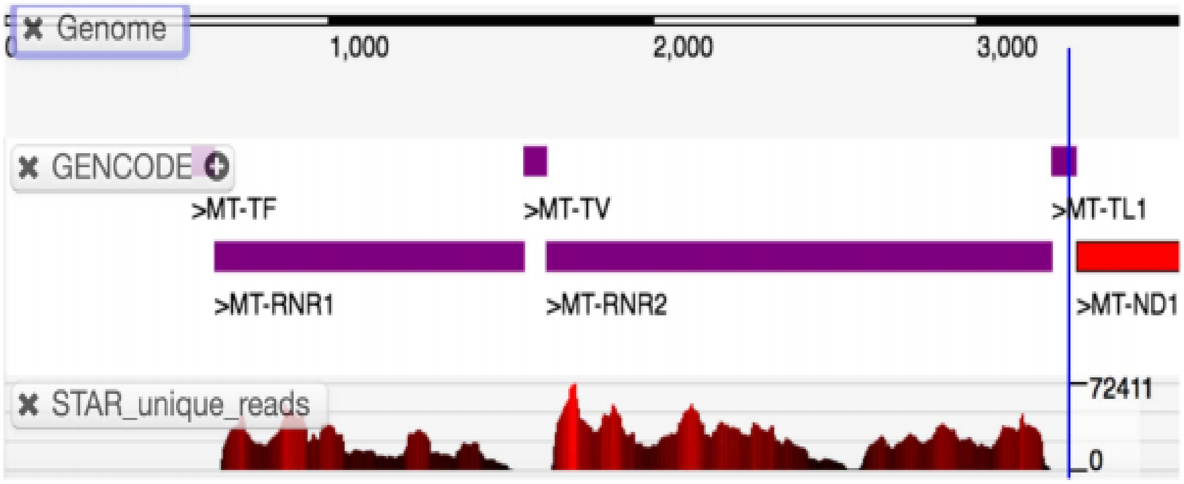

B

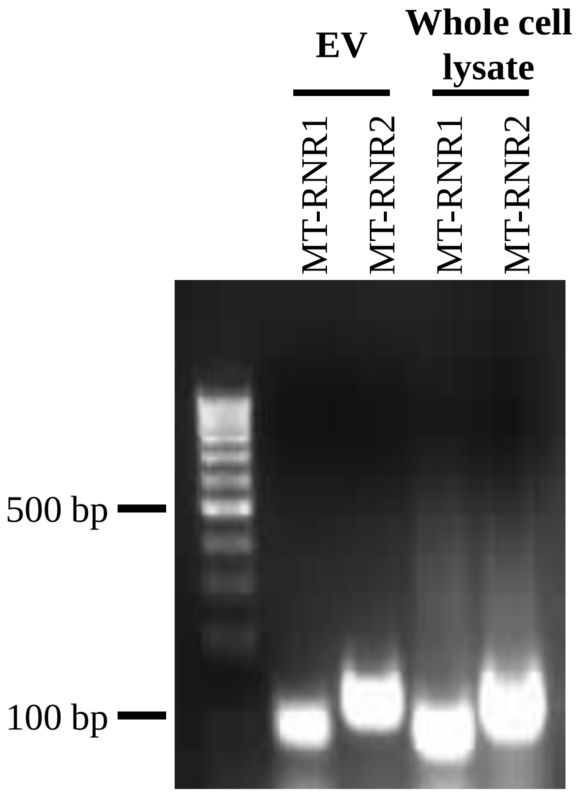

Supplementary Figure S6. (**A**) A genome browser view of reads that are aligned to MT-RNR1 and MT-RNR2, two of the most abundant transcripts found in the extracellular vesicles derived from W8B2$^+$ CSCs. (**B**) PCR analysis of MT-RNR1 and MT-RNR2 in the extracellular vesicles (EV) and whole cell lysate of W8B2$^+$ CSCs.

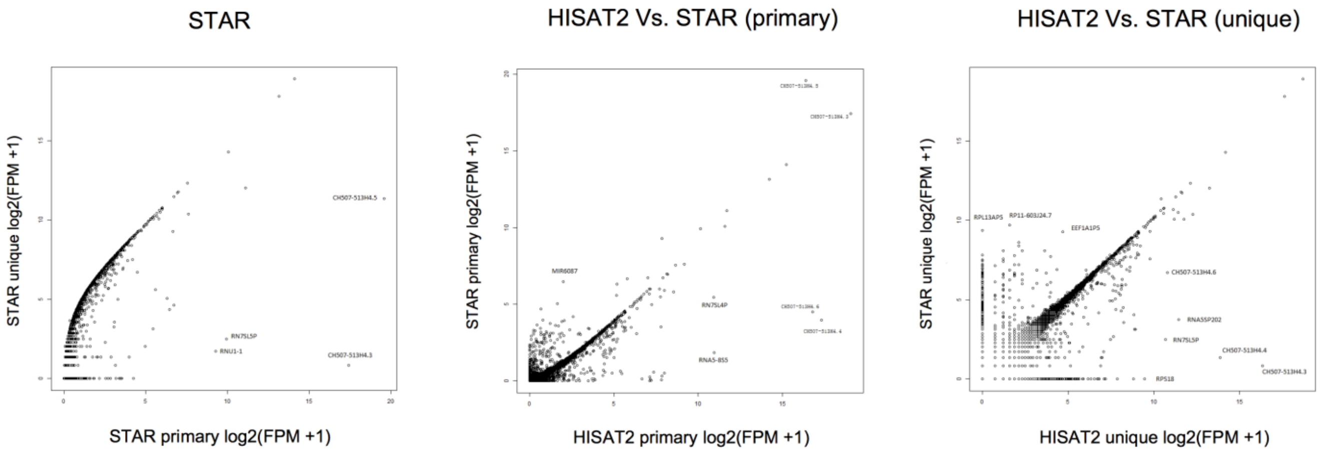

Supplementary Figure S7. Comparison of gene expression values identified based on alignment using two programs (STAR and HISAT) and two levels of stringency (Unique and Primary).

References

1 Zhang, Y. *et al.* Cardiac Repair With a Novel Population of Mesenchymal Stem Cells Resident in the Human Heart. *Stem Cells* **33** 3100-13 (2015).

2 Welton, J. L., Webber, J. P., Botos, L. A., Jones, M. & Clayton, A. Ready-made chromatography columns for extracellular vesicle isolation from plasma. *J Extracell Vesicles* **4**, 27269 (2015).

3 Paquet-Fifield, S. *et al.* Vascular endothelial growth factor-d modulates caliber and function of initial lymphatics in the dermis. *J Invest Dermatol* **133**, 2074-84 (2013).

4 Pathan, M. *et al.* FunRich: An open access standalone functional enrichment and interaction network analysis tool. *Proteomics* **15**, 2597-601 (2015).

5 Dobin, A. *et al.* STAR: ultrafast universal RNA-seq aligner. *Bioinformatics* **29**, 15-21 (2013).

6 Kim, D., Langmead, B. & Salzberg, S. L. HISAT: a fast spliced aligner with low memory requirements. *Nat Methods* **12**, 357-60 (2015).

7 Liao, Y., Smyth, G. K. & Shi, W. featureCounts: an efficient general purpose program for assigning sequence reads to genomic features. *Bioinformatics* **30**, 923-30 (2014).

8 Harrow, J. *et al.* GENCODE: the reference human genome annotation for The ENCODE Project. *Genome Res* **22**, 1760-74 (2012).

9 Reimand, J. *et al.* g:Profiler-a web server for functional interpretation of gene lists (2016 update). *Nucleic Acids Res* **44**, W83-9 (2016).

10 Agarwal, V., Bell, G. W., Nam, J. W. & Bartel, D. P. Predicting effective microRNA target sites in mammalian mRNAs. *Elife* **4** (2015).
